# Population Structure of *Geosmithia morbida*, the Causal Agent of Thousand Cankers Disease of Walnut Trees in the United States

**DOI:** 10.1371/journal.pone.0112847

**Published:** 2014-11-13

**Authors:** Marcelo M. Zerillo, Jorge Ibarra Caballero, Keith Woeste, Andrew D. Graves, Colleen Hartel, Jay W. Pscheidt, Jadelys Tonos, Kirk Broders, Whitney Cranshaw, Steven J. Seybold, Ned Tisserat

**Affiliations:** 1 Department of Bioagricultural Sciences and Pest Management, Colorado State University, Fort Collins, Colorado, United States of America; 2 USDA Forest Service Hardwood Tree Improvement and Regeneration Center, Department of Forestry and Natural Resources, Purdue University, West Lafayette, Indiana, United States of America; 3 USDA Forest Service, Forest Health Protection, Albuquerque, New Mexico, United States of America; 4 Department of Forestry and Natural Resources, Purdue University, West Lafayette, Indiana, United States of America; 5 Department of Botany and Plant Pathology, Oregon State University, Corvallis, Oregon, United States of America; 6 Department of Biological Sciences, University of New Hampshire, Durham, New Hampshire, United States of America; 7 USDA Forest Service, Pacific Southwest Research Station, Davis, California, United States of America; USDA Forest Service - RMRS, United States of America

## Abstract

The ascomycete *Geosmithia morbida* and the walnut twig beetle *Pityophthorus juglandis* are associated with thousand cankers disease of *Juglans* (walnut) and *Pterocarya* (wingnut). The disease was first reported in the western United States (USA) on several *Juglans* species, but has been found more recently in the eastern USA in the native range of the highly susceptible *Juglans nigra*. We performed a comprehensive population genetic study of 209 *G. morbida* isolates collected from *Juglans* and *Pterocarya* from 17 geographic regions distributed across 12 U.S. states. The study was based on sequence typing of 27 single nucleotide polymorphisms from three genomic regions and genotyping with ten microsatellite primer pairs. Using multilocus sequence-typing data, 197 *G. morbida* isolates were placed into one of 57 haplotypes. In some instances, multiple haplotypes were recovered from isolates collected on the same tree. Twenty-four of the haplotypes (42%) were recovered from more than one isolate; the two most frequently occurring haplotypes (H02 and H03) represented 36% of all isolates. These two haplotypes were abundant in California, but were not recovered from Arizona or New Mexico. *G. morbida* population structure was best explained by four genetically distinct groups that clustered into three geographic regions. Most of the haplotypes isolated from the native range of *J. major* (Arizona and New Mexico) were found in those states only or present in distinct genetic clusters. There was no evidence of sexual reproduction or genetic recombination in any population. The scattered distribution of the genetic clusters indicated that *G. morbida* was likely disseminated to different regions at several times and from several sources. The large number of haplotypes observed and the genetic complexity of *G. morbida* indicate that it evolved in association with at least one *Juglans* spp. and the walnut twig beetle long before the first reports of the disease.

## Introduction

**Figure 1:**
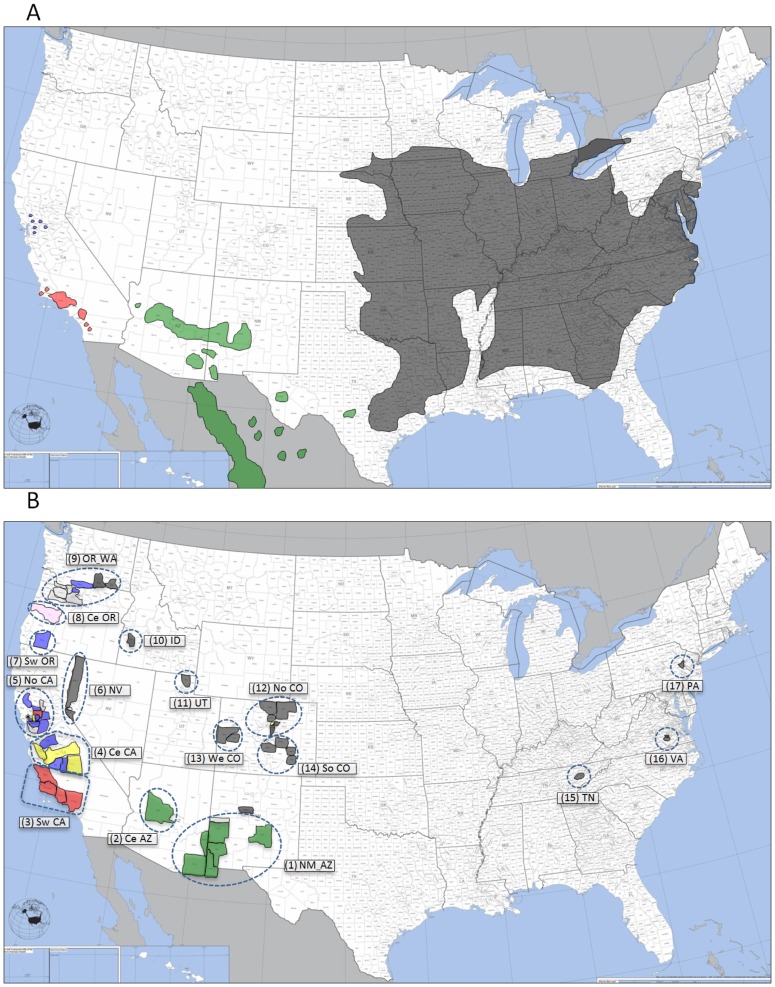
Distribution of some native species of *Juglans* in the United States (A) and sampling regions (17) of native and adventive *Juglans* for *Geosmithia morbida* (B). Regions/counties were color-coded according to the tree species with green = *J. major*; red = *J. californica*; yellow = *J. regia*; blue = *J. hindsii*; black = *J. nigra*; pink = *J. cinerea*; light gray = unidentified *Juglans* spp. or hybrids. (Figure adapted from U.S. Census Bureau and U.S. Geological Survey at https://www.census.gov/).

*Juglans nigra* L. (Juglandaceae), commonly referred to as black walnut or eastern black walnut, is a native tree species of eastern North America (Figure 1A). Its wood is highly prized for use in cabinetry, gunstocks, veneer, and other finished wood products, and the nuts are an important nutritional source for wildlife [1]–[3]. This species was widely planted in the western United States (USA) as an ornamental and nut-bearing tree [1], [2] during European colonization and the subsequent development of rural and urban landscapes. However, black walnut does not constitute a major proportion of trees in the modern urban landscape in this region.

In the early 1990 s, widespread decline and death of *J. nigra* from an undetermined cause was observed in Oregon (OR), USA [4], [5]. Similar mortality in Utah (UT) in the late 1990 s [4] and in New Mexico (NM) in 2001 [6] was attributed to drought and attack by the walnut twig beetle (WTB), *Pityophthorus juglandis* Blackman (Coleoptera: Scolytidae) [7], [8]. Beginning in 2001, decline and mortality of trees was also noted in several cities in Colorado (CO) [9]. Symptoms included yellowing of foliage and thinning of the upper crown, followed by twig and branch dieback and eventually tree death [9]. In 2008 an undescribed species of the ascomycete *Geosmithia* associated with the WTB was shown to cause bark cankers around WTB galleries in dying *J. nigra* in CO [9], and the fungus was subsequently described as *Geosmithia morbida* M. Kolařík, E. Freeland, C. Utley, & N. Tisserat sp. nov. (Ascomycota: Hypocreales) [10]. Many other *Geosmithia* species are symbionts of bark beetles, but *G. morbida* was the first phytopathogenic species reported in this genus [10]. The disease in *J. nigra* was given the common name thousand cankers disease (TCD) because of the enormous number of coalescing cankers that are formed around WTB entrance holes and galleries when the beetle aggregates in the inner bark of severely affected trees [9]. The WTB and TCD have now been documented as causing *J. nigra* mortality throughout much of the western USA and more recently, in many locations within the native range of this species [4], [11]–[18]. In 2013, TCD was also confirmed in *J. nigra* planted in northeastern Italy [19].

The source of the current TCD epidemic remains unclear. There were no recorded collections of the WTB or *G. morbida* from *J. nigra* in the western USA prior to 1959 in southern California (CA); none prior to 1996 in UT [20]; and none in the native range of *J. nigra* until 2010 [11], [12]. WTB was first collected in 1896 from *J. major* (Torr.) A. Heller (Arizona walnut) in NM [20], [21], and it has since been collected widely throughout the northern native range of this species in Arizona (AZ), NM, and once in Chihuahua in Mexico (Figure 1A) [20], [22]. *Geosmithia morbida* has also been isolated consistently from small, superficial cankers surrounding WTB galleries in native stands of *J. major* in AZ and NM, but the fungus does not cause extensive branch dieback or mortality in this species [20], [23]. This has led to speculation that the origin of the WTB and *G. morbida* is from *J. major*, and that there was a host shift in the recent past by the beetle and its fungal symbiont to the more susceptible *J. nigra*. Another possible source of the WTB and *G. morbida* in the current epidemic are populations from Los Angeles County where WTB was collected in 1959 from *J. nigra* and from *J. californica* S. Watson (southern California black walnut) [7], the latter is a native tree in this region [24]. *Geosmithia morbida* has been recovered subsequently from declining and also relatively healthy appearing *J. californica* throughout its range in southern CA (Figure 1A) [25] and from many other species of *Juglans* and the closely related *Pterocarya* growing in arboreta and germplasm collections in CA (SJS, unpublished data).

The population structure of *G. morbida* in the USA has been partially characterized. Previous studies have indicated that the fungus is genetically complex [10], [26]. Freeland and collaborators [26] identified 12 haplotypes from 145 *G. morbida* isolates collected in the western USA based on rDNA ITS sequences, and 10 haplotypes from 56 isolates based on partial β-tubulin (BT) sequence comparisons. There were no correlations among haplotypes and the hosts or geographic regions from which the isolates were collected [26]. Representative ITS haplotypes recovered from different geographic regions and hosts were all pathogenic, and although there were slight differences in aggressiveness among isolates, canker sizes were not significantly different [26]. Freeland and collaborators [26] also showed that of four *G. morbida* isolates recovered from different cankers on the same tree, all had different di-locus haplotypes based on rDNA ITS and BT sequences. Hadziabdic and collaborators [27] used 15 polymorphic microsatellite loci to reveal high haploid genetic diversity in *G. morbida* isolates collected from the eastern USA and OR. They identified two genetic clusters that corresponded to isolates collected in: 1) OR and North Carolina (NC), and 2) Pennsylvania (PA) and Tennessee (TN). A sexual state for *G. morbida* has not been detected that could account for the observed genetic variability, but clonal organisms may have limited recombination events that can change population structure [28], [29].

The main objective of our study was to determine the diversity and spatial pattern of *G. morbida* haplotypes collected widely and intensively from both native and introduced *Juglans* and *Pterocarya* hosts in various locations in the USA. *Pterocarya*, a non-native member of the Juglandaceae in the USA, was included because trees in this genus were recently reported with TCD symptoms [11]. We used multilocus sequence typing (MLST) and a microsatellite (or SSR, simple sequence repeats) analysis comprised of three genomic regions and ten markers, respectively, to observe the population structure of 209 *G. morbida* isolates collected from 17 different geographic regions (Figure 1B). Specifically we were interested in: *i*) comparing the population structure of *G. morbida* from disjunct geographic locations and ii) deducing the potential source of the TCD outbreak and whether multiple introductions of the pathogen have occurred.

## Materials and Methods

### Fungal collection and isolation

Isolates of *G*. *morbida* were collected from 54 counties in 12 states in the USA (Table 1). Samples were collected, transported, and shipped under the terms of USDA-APHIS permit P526P-11-03416. No specific permissions were required for collection of isolates from any of the locations and the field studies did not involve endangered or protected species. It is important to clarify that “population” in the manuscript means a group of geographically co-located isolates. Because *G. morbida* is primarily asexual, possibly these isolates do not represent genetically interacting entities. For the purpose of analyses, isolates were grouped into one of 17 geographic regions separated by relatively large distances: (1) NM_AZ (New Mexico and Arizona), (2) central AZ, (3) southwestern CA (California), (4) central CA, (5) northern CA, (6) NV (Nevada), (7) southwestern OR (Oregon), (8) central OR, (9) OR_WA (northern OR and southern Washington), (10) ID (Idaho), (11) UT (Utah), (12) northern CO (Colorado), (13) western CO, (14) southern CO, (15) TN (Tennessee), (16) VA (Virginia), and (17) PA (Pennsylvania) (Figure 1B and Table 1). Isolates were cultured from the margins of stem or branch cankers surrounding *P. juglandis* galleries located in the phloem or bark of symptomatic *J. californica*, *J. cinerea* L. [30], *J. hindsii* (Jeps.) Jeps. *ex* R.E. Sm., *J. major*, *J. nigra*, and *J. regia* L., from hybrids of these species, or from undetermined species of *Juglans*, and from *Pterocarya stenoptera* C. DC. (Chinese wingnut) and *P. fraxinifolia* (Lam.) Spach (Caucasian wingnut) (Figure 1B and Table 1). Isolates from *J. californica*, *J. hindsii*, *J. nigra*, and *J. regia* were collected in areas where TCD epidemics were in progress and where infected trees were readily apparent. Many *J. nigra* were large trees located in urban areas and often on private property, so sampling was restricted to trees with cankered branches that were easily accessible. Branch samples from *J. californica* and *J. hindsii* were collected from trees located in their native ranges (parks, National Forest lands, roadside riparian areas), or from germplasm collections. Samples from *J. hindsii* were also collected from roadside plantings in the Central Valley (CA) or in OR and WA. Samples from *J. regia* were collected primarily in Central Valley walnut orchards, with a few exceptions, Cache County, UT and Jefferson County, CO (Table 1). Samples from *J. major* were collected from trees located in their native range (Figure 1A), which were widely scattered, sparse and did not exhibit severe symptoms of TCD. Thus, for *J. major*, samples were collected arbitrarily from declining or asymptomatic trees that were identified during road surveys. Isolation from cankers was performed as described previously [9].

**Table 1:** Locations, hosts, haplotypes and genetic clusters based on the four-cluster-MLST-DAPC model, for *Geosmithia morbida* isolates.

Cluster	Haplotype	Isolate	GeoReg*	Year	State	County	Latitude	Longitude	Collector	Host
1	H01	1582^J^	4_CeCA	2011	CA	Merced	37°18’55.3”	−120°31’38.2”	SJS/PLD	*J. hindsii*
1	H03	1217 ** ☆	12_NoCO	2007	CO	Boulder	40°0’53”	−105°16’13”	NT	*J. nigra*
1	H03	1249	5_NoCA	2008	CA	Yolo	38°32’21.4”	−121°47’46.4”	SJS	*J. californica*
1	H03	1250	12_NoCO	2008	CO	Boulder	40°0’53”	−105°16’13”	NT	*J. nigra*
1	H03	1261	5_NoCA	2008	CA	San Joaquin	37°51’05.7”	−121°16’59.0”	SJS	*J. hindsii*
1	H03	1262	3_SwCA	2008	CA	Ventura	34°28’26.6”	−118°45’39.4”	SJS/TWC	*J. californica*
1	H03	1308^B^	12_NoCO	2009	CO	Jefferson	39°45’30.42”	−105°04’49.8”	CU	*J. nigra*
1	H03	1387	5_NoCA	2009	CA	Solano	38°29’54.4”	121°58’20.2”	SJS	*J. hindsii*
1	H03	1441	9_ORWA	2009	OR	Clackamas	45°11’24.0”	−122°12’36.0”	JP/CU	*Juglans* sp.
1	H03	1484 ^D^	5_NoCA	2010	CA	Lake	39°10’21.4”	−122°54’32.2”	SJS	*J. hindsii*
1	H03	1489 ^D^	5_NoCA	2010	CA	Lake	39°10’21.4”	−122°54’32.2”	SJS	*J. hindsii*
1	H03	1490^D^	5_NoCA	2010	CA	Lake	39°10’21.4”	−122°54’32.2”	SJS	*J. hindsii*
1	H03	1491 ^D^	5_NoCA	2010	CA	Lake	39°10’21.4”	−122°54’32.2”	SJS	*J. hindsii*
1	H03	1507^E^	15_TN	2010	TN	Knox	36°01’45.82”	−83°55’28.61”	SS	*J. nigra*
1	H03	1515	4_CeCA	2010	CA	San Benito	34°36’28.5”	−120°21’08.5”	SJS	*J. regia*
1	H03	1520 ^G^	4_CeCA	2010	CA	Fresno	36°46’03.1”	−119°56’53.0”	SJS/EF	*J. regia*
1	H03	1521 ^G^	4_CeCA	2010	CA	Fresno	36°46’03.1”	−119°56’53.0”	SJS/EF	*J. regia*
1	H03	1524	15_TN	2010	TN	Knox	35°57’38.30”	−83°55’14.66”	WC	*J. nigra*
1	H03	1583^J^	4_CeCA	2011	CA	Merced	37°18’55.3”	−120°31’38.2”	SJS/PLD	*J. hindsii*
1	H03	1659	17_PA	1011	PA	Bucks	39°57’08.41”	−75°09’49.64”	SJS	*J. nigra*
1	H03	1699	4_CeCA	2011	CA	Tulare	36°15’03.12”	−119°13’01.31”	SJS/EF	*J. hindsii*
1	H03	1822	5_NoCA	2012	CA	Solano	38°30’02.4”	−121°58’42.2”	SJS/PLD/SMH	*P. stenoptera*
1	H05	1236 **	9_ORWA	2007	WA	Benton	46°12’24.48”	−119°46’08.12”	WC	*J. nigra*
1	H08	1505	15_TN	2010	TN	Knox	35°57’38.30”	−83°55’14.66”	SJS	*J. nigra*
1	H09	1274	12_NoCO	2008	CO	Jefferson	39°45’57.95”	−105°04’37.94”	CU	*J. nigra*
1	H09	1459	5_NoCA	2010	CA	Contra Costa	37°47’00.6”	−121°58’31.8”	SJS	*J. hindsii*
1	H09	1599^K^	12_NoCO	2011	CO	Boulder	40°13’28.95”	−105°16’16.96”	NT	*J. nigra*
1	H09	1662^O^	16_VA	2011	VA	Dinwiddie	37°15’44.8”	−77°24’43.0”	SJS	*J. nigra*
1	H09	1667^O^	16_VA	2011	VA	Chesterfield	37°15’44.8”	−77°24’43.0”	SJS	*J. nigra*
1	H11	1233	5_NoCA	2008	CA	Yolo	38°32’50”	−121°47’52	SJS	*J. hindsii*
1	H11	1272	3_SwCA	2008	CA	Ventura	34°28’26.6”	−118°45’39.4”	SJS/TWC	*J. californica*
1	H11	1503	none	2010	NM	Bernalillo	35°06’38.53”	−106°36’35.97”	SJS	*J. nigra*
1	H16	1239 **	12_NoCO	2008	CO	Jefferson	39°50’11.95”	−105°02’13.94”	NT	*J. regia*
1	H16	1271	12_NoCO	2008	CO	Jefferson	39°45’19.95”	−105°13’15.96”	NT	*J. nigra*
1	H17	1573	1_NMAZ	2011	NM	Catron	33°37’10.7”	−108°53’39.1”	SJS/ADG	*J. major*
1	H18	1311 ^B^	12_NoCO	2009	CO	Jefferson	39°45’30.42”	−105°04’49.8”	CU	*J. nigra*
1	H18	1547	12_NoCO	2010	CO	Denver	39°44’24”	−104°59’32”	NT	*J. nigra*
1	H19	1273	12_NoCO	2008	CO	Jefferson	39°45’30.42”	−105°13’15.95”	CU	*J. nigra*
1	H19	1312^B^	12_NoCO	2008	CO	Jefferson	39°45’30.42”	−105°04’49.8”	CU	*J. nigra*
1	H19	1313^B^	12_NoCO	2009	CO	Jefferson	39°45’30.42”	−105°04’49.8”	CU	*J. nigra*
1	H19	1596^K^	12_NoCO	2011	CO	Boulder	40°13’28.95”	−105°16’16.96”	NT	*J. nigra*
1	H19	1597^K^	12_NoCO	2011	CO	Boulder	40°13’28.95”	−105°16’16.96”	NT	*J. nigra*
1	H19	1598^K^	12_NoCO	2011	CO	Boulder	40°13’28.95”	−105°16’16.96”	NT	*J. nigra*
1	H21	1453	1_NMAZ	2010	NM	Grant	32°37’18.7”	−108°24’25.2”	DL	*J. major*
1	H21	1540	1_NMAZ	2010	NM	Lincoln	33°44’24.0”	−105°27’36.0”	WC	*J. major*
1	H21	1558	1_NMAZ	2010	NM	Grant	32°43’48.0”	−108°22’48.0”	WC	*J. major*
1	H21	1559	1_NMAZ	2010	NM	Grant	32°43’48.0”	−108°22’48.0”	WC	*J. major*
1	H21	1670	1_NMAZ	2011	NM	Lincoln	33°06’22.5”	−105°48’17.8”	ADG	*J. major*
1	H22	1478^C^	3_SwCA	2010	CA	Santa Barbara	34°36’28.5”	−120°21’08.5”	SJS	*J. californica*
1	H23	1309	11_UT	2009	UT	Cache	41°55’41.91”	−111°48’29.33”	NT	*J. nigra*
1	H25	1462	5_NoCA	2010	CA	Contra Costa	37°47’00.6”	−121°58’31.8”	SJS	*J. hindsii*
1	H25	1476 ^C^	3_SwCA	2010	CA	Santa Barbara	34°36’28.5”	−120°21’08.5”	SJS	*J. californica*
1	H25	1518^F^	4_CeCA	2010	CA	Fresno	36°46’03.1”	−119°56’53.0”	SJS/EF	*J. regia*
1	H26	1407	4_CeCA	2009	CA	Tulare	36°14’08.6”	−119°14’32.6”	SJS/ADG/EF	*J. regia*
1	H26	1461	5_NoCA	2010	CA	Contra Costa	37°47’00.6”	−121°58’31.8”	SJS	*J. hindsii*
1	H26	1486 ^D^	5_NoCA	2010	CA	Lake	39°10’21.4”	−122°54’32.2”	SJS	*J. hindsii*
1	H27	1440	9_ORWA	2009	OR	Clackamas	45°11’24.0”	−122°12’36.0”	JP/CU	*Juglans* sp.
1	H27	1509^E^	15_TN	2010	TN	Knox	36°01’45.82”	−83°55’28.61”	SJS	*J. nigra*
1	H29	1506 ^E^	15_TN	2010	TN	Knox	36°01’45.82”	−83°55’28.61”	SJS	*J. nigra*
1	H35	1545	12_NoCO	2010	CO	Denver	39°44’24”	−104°59’32”	NT	*J. nigra*
1	H43	1572^H^	1_NMAZ	2011	NM	Hidalgo	31°26’25.6”	−108°58’45.9”	SJS/ADG	*J. major*
1	H44	1314^B^	12_NoCO	2009	CO	Jefferson	39°45’30.42”	−105°04’49.8”	CU	*J. nigra*
1	H56	1821^P^–	5_NoCA	2012	CA	Solano	38°19’10”	−121°55’27”	SJS/PLD/SMH	*P. stenoptera*
2	H02	1224	12_NoCO	2008	CO	Boulder	40°13’28”	−105°16’16”	NT	*J. nigra*
2	H02	1225	12_NoCO	2008	CO	Boulder	40°13’28”	−105°16’16”	NT	*J. nigra*
2	H02	1227	5_NoCA	2009	CA	Yolo	38°32’21.4”	−121°47’46.4”	SJS	*J. californica*
2	H02	1245	10_ID	2008	ID	Ada	43°35’56.76”	−116°12’52.58”	WC	*J. nigra*
2	H02	1252	3_SwCA	2008	CA	S Luis Obispo	35°03’08.0”	−119°54’33.0”	SJS/DLW	*J. hindsii* X *J. major*
2	H02	1260	9_ORWA	2008	OR	Hood River	45°42’24.1”	−121°31’18.1”	JP/CU	*Juglans* sp.
2	H02	1263	3_SwCA	2008	CA	Ventura	34°28’26.6”	−118°45’39.4”	SJS/TWC	*J. californica*
2	H02	1264	3_SwCA	2008	CA	Ventura	34°28’25.8”	−118°45’39.4”	SJS/TWC	*J. californica*
2	H02	1267	5_NoCA	2008	CA	San Joaquin	37°51’05.7”	−121°16’59.0”	SJS	*J. hindsii*
2	H02	1268	3_SwCA	2008	CA	Ventura	34°20’02.5”	−118°54’07.5”	SJS/TWC	*J. californica*
2	H02	1270	5_NoCA	2008	CA	San Joaquin	37°51’05.7”	−121°16’59.0”	SJS	*J. hindsii*
2	H02	1275	3_SwCA	2008	CA	Ventura	34°20’02.5”	−118°54’07.5”	SJS/TWC	*J. californica*
2	H02	1279	12_NoCO	2008	CO	Jefferson	39°45’30.42”	−105°04’49.8”	CU	*J. nigra*
2	H02	1289	5_NoCA	2009	CA	Yolo	38°32’21.4”	−121°47’46.4”	SJS	*J. californica*
2	H02	1315^B^	12_NoCO	2009	CO	Jefferson	39°45’30.42”	−105°04’49.8”	CU	*J. nigra*
2	H02	1324	12_NoCO	2009	CO	Boulder	40°03’05”	−105°02’59”	CU	*J. nigra*
2	H02	1334	14_SoCO	2009	CO	Otero	38°03’09”	−103°43’12”	WC	*J. nigra*
2	H02	1335	14_SoCO	2009	CO	Crowley	38°09’55”	−103°56’46”	WC	*J. nigra*
2	H02	1345	9_ORWA	2009	OR	Hood River	45°42’24.1”	−121°31’18.1”	JP/CU	*Juglans* sp.
2	H02	1352	3_SwCA	2009	CA	Los Angeles	34°03’02.5”	−117°49’29.7”	SJS	*J. californica*
2	H02	1355	5_NoCA	2009	CA	Yolo	38°32’21.4”	−121°47’46.4”	SJS	*J. hindsii*
2	H02	1365	5_NoCA	2009	CA	Yolo	38°32’21.4”	−121°47’46.4”	SJS	*J. hindsii*
2	H02	1381	9_ORWA	2009	OR	Clackamas	45°11’24.0”	−122°12’36.0”	JP/CU	*Juglans* sp.
2	H02	1385	5_NoCA	2009	CA	Sutter	39°03’40.9”	−121°36’49.1	SJS	*J. nigra* X *J. hindsii*
2	H02	1392	5_NoCA	2009	CA	Yolo	38°32’05.0”	−121°48’12.1”	SJS	*J. hindsii*
2	H02	1403	4_CeCA	2009	CA	Kings	36°19’41.0”	−119°32’37.4”	SJS/ADG	*J. hindsii*
2	H02	1408	12_NoCO	2009	CO	Boulder	40°13’28”	−105°16’16”	NT	*J. nigra*
2	H02	1427	5_NoCA	2009	CA	Sutter	39°03’40.9”	−121°36’49.1”	SJS	*J. nigra X J. hindsii*
2	H02	1452	5_NoCA	2009	CA	Yolo	38°32’21.4”	−121°58’14.2”	SJS	*J. hindsii*
2	H02	1481^C^	3_SwCA	2010	CA	Santa Barbara	34°36’28.5”	−120°21’08.5”	SJS	*J. californica*
2	H02	1482 ^C^	3_SwCA	2010	CA	Santa Barbara	34°36’28.5”	−120°21’08.5”	SJS	*J. californica*
2	H02	1485 ^D^	5_NoCA	2010	CA	Lake	39°10’21.4”	−122°54’32.2”	SJS	*J. hindsii*
2	H02	1493	3_SwCA	2010	CA	Los Angeles	34°03’12”	−118°14’43”	WC	*J. californica*
2	H02	1514	4_CeCA	2009	CA	Tulare	36°14’08.6”	−119°14’32.6”	SJS/ADG/EF	*J. regia*
2	H02	1523	15_TN	2010	TN	Knox	35°55’30.05”	−83°59’19.85”	SJS/CL	*J. nigra*
2	H02	1525	15_TN	2010	TN	Knox	35°55’30.05”	−83°59’19.85”	SJS/CL	*J. nigra*
2	H02	1530	9_ORWA	2010	WA	Klickitat	45°44’49.9”	−120°26’16.2”	SJS/CL	*J. hindsii*
2	H02	1535	14_SoCO	2010	CO	Otero	38°03’9”	−103°43’12”	WC	*J. nigra*
2	H02	1581^J^	4_CeCA	2011	CA	Merced	37°18’55.3”	−120°31’38.2”	SJS/PLD	*J. hindsii*
2	H02	1590	9_ORWA	2011	WA	Walla Walla	46°13’48.0”	−118°28’48.0”	JM	*J. nigra*
2	H02	1600^K^	12_NoCO	2011	CO	Boulder	40°13’28.95”	−105°16’16.96”	NT	*J. nigra*
2	H02	1602^K^	12_NoCO	2011	CO	Boulder	40°13’28.95”	−105°16’16.96”	NT	*J. nigra*
2	H02	1631^M^	6_NV	2011	NV	Carson City	39°10’21.55”	−119°46’7.92”	ADG/TWC	*Juglans* sp.
2	H02	1633^M^	6_NV	2011	NV	Carson City	39°10’21.55”	−119°46’7.92”	ADG/TWC	*Juglans* sp.
2	H02	1636^N^	3_SwCA	2011	CA	Los Angeles	34°03’12”	−103°56’46”	WC	*J. californica*
2	H02	1660	16_VA	2011	VA	Dinwiddie	37°15’44.8”	−77°24’43.0”	SJS	*J. nigra*
2	H02	1665^O^	16_VA	2011	VA	Chesterfield	37°15’44.8”	−77°24’43.0”	SJS	*J. nigra*
2	H02	1704	6_NV	2011	NV	Washoe	39°31’13.73”	−119°48’27.9”	SJS/PLD	*J. nigra*
2	H02	1830^P^	35_NoCA	2012	CA	Solano	38°19’10”	−121°55’27”	SJS/PLD/SMH	*P. fraxinifolia*
2	H02	2032	22_SwCA	2014	CA	Los Angeles	34°8.452’	−118°3.444’	KJG/JEH/SMH/AL/LMO	*P. fraxinifolia*
2	H04	1223	11_UT	2008	UT	Cache	41°55’1.74”	−111°48’48.8”	NT	*J. nigra*
2	H04	1266	3_SwCA	2008	CA	Ventura	34°25.548’	−119°05.354’	SJS/TWC	*J. californica*
2	H04	1338	9_ORWA	2009	OR	Marion	4°4’22.69”	−122°51’32.1”	JP/CU	*Juglans* sp.
2	H04	1368	13_WeCO	2009	CO	Mesa	39°3’49.93”	−108°33’2.3”	BH	*J. nigra*
2	H04	1380	5_NoCA	2009	CA	Sacramento	38°39.6624’	−121 28.723’	SJS	*J. hindsii*
2	H04	1432	9_ORWA	2009	OR	Wasco	45°09’36.0”	−121°09’36.0”	JP/CU	*Juglans* sp.
2	H04	1439	9_ORWA	2009	OR	Marion	45°8’ 32.17”	−122°51’ 32.1”	JP/CU	*Juglans* sp.
2	H04	1534	7_SwOR	2010	OR	Jackson	42°25’37.6”	−122°57’20.5”	SJS/CL	*J. hindsii*
2	H06	1366	1_NMAZ	2009	AZ	Yavapai	34°58’25.1”	−112°39’52.4”	WC	*J. major*
2	H07	1428	5_NoCA	2010	CA	Alameda	37°40’29.3”	−121°53’8.9”	SJS/PLD	*J. hindsii*
2	H10	1258	9_ORWA	2008	OR	Wasco	45°36’04.0”	−121°10’58.1”	MP	*Juglans* sp.
2	H10	1276	12_NoCO	2008	CO	Larimer	40°18’22”	−105°4’44.9”	NT	*J. nigra*
2	H10	1346	9_ORWA	2009	OR	Wasco	45°41’1.14”	−121°23’51.9”	JP/CU	*Juglans* sp.
2	H10	1601^K^	12_NoCO	2011	CO	Boulder	40°13’28.95”	−105°16’16.96”	NT	*J. nigra*
2	H10	1672	15_TN	2011	TN	Knox	35°55’30.05”	−83°59’19.85”	SJS/CL	*J. nigra*
2	H12	1533	7_SwOR	2010	OR	Jackson	42°25’37.6”	−122°57’20.5”	SJS/CL	*J. hindsii*
2	H12	1584^J^	4_CeCA	2010	CA	Merced	37°18’55.3”	−120°31’38.2”	SJS/PLD	*J. hindsii*
2	H13	1393	12_NoCO	2009	CO	Weld	40°18’ 9 “	−105°4’51”	NT	*J. nigra*
2	H14	1256 **	9_ORWA	2008	OR	Multnomah	45°30’42.48”	−122°40’32.2”	MP	*Juglans* sp.
2	H14	1634^N^	3_SwCA	2011	CA	Los Angeles	34°3’ 12”	−103°56’ 46”	WC	*J. californica*
2	H15	1610	5_NoCA	2011	CA	Yolo	38°32’21.4”	−121°47’42.0”	SJS/CL	*J. major* X *J. hindsii*
2	H20	1285	13_WeCO	2008	CO	Delta	38°44’ 41.316”	−108°4’ 15.0”	BH	*J. nigra*
2	H20	1305^B^	12_NoCO	2009	CO	Jefferson	39°45’30.42”	−105°04’49.8”	CU	*J. nigra*
2	H24	1218 ** ☆	12_NoCO	2007	CO	Boulder	40°0’56”	−105°16’45”	NT	*J. nigra*
2	H24	1259	9_ORWA	2008	OR	Marion	45°08’49.9”	−122°51’29.9”	JP/CU	*Juglans* sp.
2	H24	1318	14_SoCO	2009	CO	Otero	38°03’11.56”	−103°42’52.7”	WC	*J. nigra*
2	H24	1404	4_CeCA	2009	CA	Tulare	36°14’08.6”	−119°14’32.6”	SJS/ADG/EF	*J. regia*
2	H24	1494	3_SwCA	2011	CA	Los Angeles	34°03’12”	−103°56’46”	WC	*J. californica*
2	H24	1532	9_ORWA	2010	OR	Wasco	45°36’29.6”	−121°07’40.3”	SJS/CL	*J. hindsii*
2	H28	1409	4_CeCA	2009	CA	Tulare	36°14’08.6”	−119°14’32.6”	SJS/ADG/EF	*J. regia*
2	H30	1269**	3_SwCA	2008	CA	Ventura	34°20’02.5”	−118°54’07.5”	SJS/TWC	*J. californica*
2	H30	1350	12_NoCO	2009	CO	Jefferson	39°49’12.0”	−105°06’40.0”	CU	*J. nigra*
2	H30	1430	9_ORWA	2009	OR	Marion	45°08’32.14”	−122°51’31.8”	JP/CU	*Juglans* sp.
2	H30	1519^G^	4_CeCA	2010	CA	Fresno	36°46’03.1”	−119°56’53.0”	SJS/EF	*J. regia*
2	H30	1522	15_TN	2010	TN	Knox	35°57’37.76”	−83°55’15.2”	WC/NT	*J. nigra*
2	H30	1726	8_CeOR	2011	OR	Lane	43°57’00.0”	−122°52’48.0”	MS	*J. cinerea*
2	H30	1727	8_CeOR	2011	OR	Lane	43°57’00.0”	−122°52’48.0”	MS	*J. cinerea*
2	H31	1483 ^D^	5_NoCA	2010	CA	Lake	39°10’21.4”	−122°54’32.2”	SJS	*J. hindsii*
2	H34	1247	14_SoCO	2008	CO	El Paso	38°50’0.35”	−104°49’18.5”	NT	*J. nigra*
2	H42	1623^L^	1_NMAZ	2011	AZ	Cochise	31°35’32.5”	−109°30’23.9”	TWC	*J. major*
2	H42	1624^L^	1_NMAZ	2011	AZ	Cochise	31°35’32.5”	−109°30’23.9”	TWC	*J. major*
2	H42	1627^L^	1_NMAZ	2011	AZ	Cochise	31°35’32.5”	−109°30’23.9”	TWC	*J. major*
2	H45	1626^L^	1_NMAZ	2011	AZ	Cochise	31°35’32.5”	−109°30’23.9”	TWC	*J. major*
2	H46	1628^L^	1_NMAZ	2011	AZ	Cochise	31°35’32.5”	−109°30’23.9”	TWC	*J. major*
2	H47	1402	9_ORWA	2009	OR	Wasco	45°41’1.14”	−121°23’51.9”	JP	*Juglans* sp.
2	H47	1585	4_CeCA	2011	CA	Tulare	36°07’51.1”	−119°08’16.9”	SJS/EF/PLD	*J. regia*
2	H47	1586	4_CeCA	2011	CA	Tulare	36°07’51.1”	−119°08’16.9”	SJS/EF/PLD	*J. regia*
2	H48	1306^B^	12_NoCO	2009	CO	Jefferson	39°45’30.42”	−105°04’49.8”	CU	*J. nigra*
2	H49	1496	14_SoCO	2010	CO	Fremont	38°26’26.34”	−105°14’17.3”	WC	*J. nigra*
2	H51	1708	6_NV	2011	NV	Douglas	39°0’15.10”	−119°50’42.0”	SJS/PD/GD	*J. nigra*
2	H52	1625^L^	1_NMAZ	2011	AZ	Cochise	31°35’32.5”	−109°30’23.9”	TWC	*J. major*
2	H55	1513	5_NoCA	2010	CA	Solano	38°16’12.0”	−121°56’24.0”	RMB	*J. regia*
2	H57	1246	14_SoCO	2008	CO	El Paso	38°50’0.35”	−104°49’18.5”	NT	*J. nigra*
3	H36	1569^H^	1_NMAZ	2011	NM	Hidalgo	31°26’25.6”	−108°58’45.9”	SJS/ADG	*J. major*
3	H37	1576^I^	1_NMAZ	2011	NM	Catron	33°37’10.7”	−108°53’39.1”	SJS/ADG	*J. major*
3	H38	1301 ^B^	12_NoCO	2009	CO	Jefferson	39°45’30.42”	−105°04’49.8”	CU	*J. nigra*
3	H38	1302 ^A^	2_CeAZ	2009	AZ	Yavapai	34°55’25.1”	−112°50’34.7”	WC	*J. major*
3	H38	1321 ^B^	12_NoCO	2009	CO	Jefferson	39°45’30.42”	−105°04’49.8”	CU	*J. nigra*
3	H38	1340	1_NMAZ	2009	NM	Grant	32°43’48.0”	−108°22’48.0”	WC	*J. major*
3	H38	1388	1_NMAZ	2009	AZ	Yavapai	34°55’25.1”	−112°50’34.7”	WC	*J. major*
3	H38	1401	1_NMAZ	2009	NM	Catron	33°37’10.7”	−108°53’39.1”	SJS/ADG	*J. major*
3	H38	1554	2_CeAZ	2010	AZ	Yavapai	34°41’42.62”	−112°09’44.4”	WC	*J. major*
3	H38	1556	1_NMAZ	2010	NM	Grant	32°38’17.9”	−108°06’18.4”	WC	*J. major*
3	H38	1574^I^	1_NMAZ	2010	NM	Catron	33°37’10.7”	−108°53’39.1”	SJS/ADG	*J. major*
3	H39	1373	1_NMAZ	2010	NM	Grant	32°54’21.7”	−107°43’45.5”	DL	*J. major*
3	H39	1421	1_NMAZ	2010	NM	Grant	32°54’21.7”	−107°43’45.5”	DL	*J. major*
3	H39	1422	1_NMAZ	2010	NM	Grant	32°55’08.5”	−107°34’35.1”	DL	*J. major*
3	H39	1557	1_NMAZ	2010	NM	Grant	32°52’44.5”	−107°52’04.5”	WC	*J. major*
3	H40	1248	12_NoCO	2008	CO	Jefferson	39°45’30.42”	−105°13’15.95”	NT	*J. nigra*
3	H40	1322	2_CeAZ	2009	AZ	Yavapai	34°27’05.32”	−112°16’09.39”	WC	*J. major*
3	H40	1323^B^	12_NoCO	2008	CO	Jefferson	39°45’30.42”	−105°04’49.8”	NT	*J. nigra*
3	H40	1358	2_CeAZ	2009	AZ	Yavapai	34°55’25.1”	−112°50’34.7”	WC	*J. major*
3	H40	1420	2_CeAZ	2009	AZ	Yavapai	34°55’25.1”	−112°50’34.7”	WC	*J. major*
3	H41	1571^H^	1_NMAZ	2010	NM	Hidalgo	31°26’25.6”	−108°58’45.9”	SJS/ADG	*J. major*
3	H50	1644	15_TN	2011	TN	Knox	35°55’22.8”	−83°59’31.4”	SF	*J. nigra*
3	H53	1479^C^	3_SwCA	2010	CA	Santa Barbara	34°36’28.5”	−120°21’08.5”	SJS	*J. californica*
3	H54	1570 ^H^	1_NMAZ	2010	NM	Hidalgo	31°26’25.6”	−108°58’45.9”	SJS/ADG	*J. major*
4	H32	1299** ^A^	2_CeAZ	2009	AZ	Yavapai	34°55’25.1”	−112°50’34.7”	WC	*J. major*
4	H33	1234 **	2_CeAZ	2008	AZ	Yavapai	34°55’25.1”	−112°50’34.7”	WC	*J. major*
4	H33	1303 ^A^	2_CeAZ	2009	AZ	Yavapai	34°55’25.1”	−112°50’34.7”	WC	*J. major*
4	H33	1307 ^A^	2_CeAZ	2009	AZ	Yavapai	34°55’25.1”	−112°50’34.7”	WC	*J. major*
4	H33	1412	1_NMAZ	2009	AZ	Yavapai	34°55’25.1”	−112°50’34.7”	WC	*J. major*
4	H33	1550	2_CeAZ	2010	AZ	Yavapai	34°55’25.1”	−112°50’34.7”	WC	*J. major*
4	H33	1675	2_CeAZ	2010	AZ	Yavapai	34°55’25.1”	−112°50’34.7”	WC	*J. major*
–	–	1222	11_UT	2007	UT	Cache	41°55’1.74”	−111°48’48.81”	NT	*J. regia*
–	–	1228	5_NoCA	2008	CA	Yolo	38°32’21.4”	−121°47’46.4”	SJS	*J. californica*
–	–	1229	5_NoCA	2008	CA	Yolo	38°32’21.4”	−121°47’46.4”	SJS	*J. californica*
–	–	1347	OR_WA	2009	OR	Wasco	45°41’0.43”	−121°23’50.26”	JP	*Juglans* sp.
–	–	1348	3_SwCA	2009	CA	Los Angeles	34°30’48.2”	−118°37’04.6”	SJS	*J. californica*
–	–	1349	9_ORWA	2009	OR	Wasco	45°41’0.43”	−121°23’50.26”	JP	*Juglans* sp.
–	–	1359	13_WeCO	2009	CO	Mesa	39°03’49.93”	−108°33’2.33”	BH	*J. nigra*
–	–	1383	3_SwCA	2009	CA	Los Angeles	34°30’48.2”	–118°37’04.6”	SJS	*J. californica*
–	–	1405	4_CeCA	2009	CA	Kings	36°19’41.0”	−119°32’37.4”	SJS/ADG	*J. hindsii*
–	–	1473 ^C^	3_SwCA	2010	CA	Santa Barbara	34°36’28.5”	−120°21’08.5”	SJS	*J. californica*
–	–	1528	9_ORWA	2010	WA	Walla Walla	46°03’50.8”	−118°18’40.7”	SJS/CL	*J. nigra*
–	–	1529	9_ORWA	2010	WA	Walla Walla	46°03’50.8”	−118°18’40.7”	SJS/CL	*J. nigra*

* Geographical regions are depicted in Fig. 1B.

** Isolates selected in the first trial of MLST analysis.

☆ Isolates deposited at Centraalbureau voor Schimmelcultures as CBS 124663 (1217) and CBS 124664 (1218).

Isolates underlined were subjected to SSR analysis.

A-P Common letters indicate isolates from different cankers of the same tree.

Collectors are as follows: R. M. Bostock (RMB), T. W. Coleman (TWC), W. Cranshaw (WC), P.L. Dallara (PLD), G. Durham (GD), E. Fichtner (EF), S. Fraedrich (SF), A. D. Graves (ADG), K.J. Greby (KJG), B. Hammon (BH), J.E. Henrich (JEH), S.M. Hishinuma (SMH), D. Leatherman (DL), C. Leslie (CL), A. Liu (AL), J. McKenna (JM), L.M. Ohara (LMO), J. Pscheidt (JP), M. Putnam (MP), S. Schlarbaum (SS), S. J. Seybold (SJS), N. Tisserat (NT), C. Utley (CU), D.L. Wood (DLW). All isolations were made in the laboratory of NT with the exception of isolate 1513, which was made in the laboratory of RMB.

In order to obtain a haploid individual of *G. morbida*, all isolates were either single spored by serial dilution plating of conidia or by inducing the yeast phase by rapid shaking (250 rpm) of liquid culture, followed by serial dilution plating. A total of 209 haploid isolates were maintained on one-half strength potato dextrose agar (½ PDA, Difco Corp., Sparks, MD, USA) [10]. Of these isolates, 197 or 107 were assessed by MLST or SSR analysis, respectively, whereas 95 were tested by both methods (Table 1). In some cases, multiple *G. morbida* isolates were collected from different cankers on the same tree, or from more than one *Juglans* species from the same location (Table 1) to ascertain whether individual trees or adjacent trees contained more than one *G. morbida* haplotype.

### DNA extraction

Single-spore isolates were grown on half-strength potato dextrose broth (Difco) for 3 days at 25°C with shaking (120 rpm). Mycelium was collected and lyophilized and DNA extraction was performed by using the Easy DNA Kit (Invitrogen, Carlsbad, CA, USA) or DNeasy Blood & Tissue Kit (Qiagen, Valencia, CA), according to the manufacturer’s recommendations. DNA concentrations and purities were estimated with a spectrophotometer (Thermo Scientific NanoDrop 1000, Loughborough, UK).

### Isolation and identification of SSR sequences

DNA was extracted from two single-spore-derived cultures of isolates 1217 (CBS 124663) and 1218 (CBS 124664) (Table 1), known *a priori* to be characterized by different ITS sequences. DNA from both isolates was pooled and sent to the Cornell University Genomics Facility; enzymatically digested with *Hin*cII; ligated; and enriched for microsatellites by hybridization to probes containing random repeats. Enriched fragments were processed into a mate-pair library and sequenced with 454 GS FLX (Roche, Indianapolis, IN, USA) sequencing technology. Sequences were assembled by using SeqMan Pro (Lasergene version 8.1.1; DNASTAR, Madison, WI, USA) yielding a total of 13,392 contigs and 21,737 singlets that were screened for the presence of microsatellite repeats by using EagleView software [31]. Primers were designed from a subset of selected contigs based on: presence of microsatellite repeats; putative presence of polymorphism within a contig; location of repeats within the sequence; and read quality. The software Primer 3 [32] was used to identify primer pairs from contigs. Primer pairs that produced a strong and consistent signal and produced polymorphic amplicons were selected and used to characterize DNA from designated isolates (Table 1, underlined isolates). Sequence data from loci used in this study were deposited in GenBank (http://www.ncbi.nlm.nih.gov/genbank/) (Table S1). For all loci, polymerase chain reaction (PCR) protocol was 4 min denaturation at 94°C, followed by 25 cycles of 45 sec at 94°C, 45 sec at 56°C and 45 sec at 72°C, followed by 8 cycles of 10 sec at 94°C, 45 sec at 51°C and 45 sec at 72°C. At the end of the amplification, the samples were held at 72°C for 11 min and then at 4°C until they could be removed and stored at −20°C for dilution and mixing with ROX standards. Reactions included dNTP (0.3 mM each), bovine serum albumin (10 µg/µl) (New England Biolabs, Ipswich, MA, USA), in a final volume of 15 µl and all other reagents as described previously [33]. Microsatellite haplotypes were produced by using the three primer method [33], and alleles were binned by using Gene Mapper software (Applied Biosystems, South San Francisco, CA, USA). Quality control of microsatellite markers was guaranteed by genotyping the loci of all samples at least twice and independently. All genotyping plates contained DNA from three isolates (1217, 1218, and 1234), which were used as control (allelic standards). Locus/isolate combinations that produced inconsistent allele sizes or were null were considered as missing data. The ten amplicon sizes (corresponding to each SSR) of each isolate were entered into an Excel spreadsheet and compared visually (manually). Isolates sharing the same amplicon sizes were considered to be clonal.

### Isolation and identification of MLST sequences

We attempted to amplify DNA from 16 genomic regions by using the following primer pairs: for housekeeping genes identified in the *G. morbida* genome; for those developed for *Fusarium solani* MLST analysis [34]; for the rRNA internal transcribed spacer (ITS) region [35]; and for the β-tubulin (BT) gene [36], to obtain a MLST-based analysis of *G. morbida* (Table 2). Details on primer design for MLST sequences based on the *G. morbida* genome are provided in File S1.

**Table 2:** Primers tested on MLST analysis of *Geosmithia morbida* isolates.

Gene/region	Sequence (5′ → 3′)	Genome	Outcome
ITS	1F: CTTGGTCATTTAGAGGAAGTAA 4: TCCTCCGCTTATTGATATGC	Fungi	single band and SNPs
BT	1: AACATGCGTGAGATTGTAAGT Geo*: TCTCGACAAAACGTACCTCGT	*Fusarium*	single band and SNPs
FsACC	F: CTCGTGAGATCATGATCCAGT R: GTTGATAACAGCGGAGAGCT	*Fusarium*	multiple unspecific band
FsGPD	F: CATGTACGTCGTCGGTGTCA R: CGCTTACTTGGAGGCATCG	*Fusarium*	multiple unspecific band
FsHMG	F: GGCAAGATTCCTGGTTACGC R: TTCATACCCATAGCGTCACC	*Fusarium*	did not amplify
FsICL	F: GGAGGTTGAGGCTGTCAAG R: GCTTGGTGAGCTTCATGACA	*Fusarium*	did not amplify
FsMPD	F: CGTCGAGAACACCATCACAAA R: ATGGGGGTTGCCAATTCGCT	*Fusarium*	did not amplify
FsSOD	F: TGGGACATCACCGGTAACGA R: CAGTCTTGAGAGACTCCTCG	*Fusarium*	did not amplify
FsTOP1	F: AGGAGCACATGACGACCAAG R: GATCCTGATCAGCCATGATC	*Fusarium*	multiple unspecific band
FsUGP1	F: CAGATGCGAAATGCTCTGAC R: AGGATATCGACGTTGTGGC	*Fusarium*	single band
Methionine aminopeptidase (MAP)	F: GCGAATAACGCTGCAATTCT R: AACCCGGAGTGACAACTGAC	*G. morbida*	single band and SNPs
Ribosomal L18ae protein family	F: CTTGGTGTTCTGCTTGGTGA R: ACCCCGAGAAGGTCAAGAAC	*G. morbida*	single band
Dolichyl-phosphate-mannose-protein mannosyltransferase	F: TCTTCTGGCTGTTCATGACG R: CGAGGACACGGAAAATGAAG	*G. morbida*	single band
Amino acid permease	F: TATCAGCGCTTGCAAATACG R: GCAATCATGGAAATGTGTCG	*G. morbida*	single band
40S ribosomal protein S2	F: GCCCATCAAGGAGTACCAGA R: GACGTGTAGGCGTCTTCGAT	*G. morbida*	single band
Kinesin	F: GCTTCGCTACAGGTGAGTCC R: AGACTCCAGCGGTTGTCCTG	*G. morbida*	single band

* BT Geo was designed based on *G. morbida* genome, inwardly oriented after amplification by using BT22 [36].

Amplification of these 16 regions by PCR consisted of 10X Standard *Taq* Reaction Buffer (New England Biolabs), 0.2 U of *Taq* DNA Polymerase, 2 mM MgCl_2_, 0.2 mM of each dNTP, 0.25 µM of each primer, 20 ng of DNA, and sterile deionized water added for a final volume of 20 µl. Annealing temperatures were initially set 5°C below the lowest T_m_ of the primer pairs. Because some of the primers were not based on the *G. morbida* genome, we also performed gradient PCR with annealing temperatures ±5°C of those set initially. Parameters were: 95°C for 5 min; followed by 35 cycles of 95°C for 30 sec, varying annealing temperatures based on the primer pair for 45 sec, 72°C for 1.5 min; and completed with 72°C for 5 min. The presence of amplicons was verified by using a Sub-Cell GT Cell (BioRad, Hercules, CA, USA) electrophoresis system. Amplified sequences that exhibited a single well-defined band were purified by using PureLink PCR Purification Kit (Invitrogen). Ten nanograms of DNA per 100 base pairs (bp) were combined with 10 pmol of primer and sequenced with the BigDye version 3.1 ready reaction kit (Applied Biosystems) on an ABI 3730 automated sequencer at the Proteomics and Metabolomics Facility at Colorado State University. Chromatograms were visualized by using Sequence Scanner Software 1.0 from Applied Biosystems (http://www.appliedbiosystems.com/) and bases were only accepted if the Phred quality score was equal to or greater than 20 [37]. Low quality reads and those containing overlapping sequences were either re-sequenced or sequenced with the primer flanking the opposite side. Sequences were edited and aligned by using BioEdit [38]. Only three of the 16 primer sets amplified genomic regions with SNPs. These resulted in 7, 13, and 7 polymorphisms in the ITS, BT, and methionine aminopeptidase (MAP) sequences (Figure S1) of 197 *G. morbida* isolates, respectively (Table 2). PCR amplicons of the ITS, BT, and MAP region/genes were 565, 438, and 468 bp in length and the sequences were trimmed to 516, 363, and 365 bp, respectively, for MLST analysis. Sequences were deposited in GenBank with the following accession numbers: ITS, KJ148225 to KJ148419; BT, KJ148030 to KJ148224; and MAP, KJ148420 to KJ148614.

### Data Analyses

#### Genetic structure and host specificity

Haplotypes (isolates in the case of SSR data) were placed into genetic clusters based on the posterior probability of their allele frequencies by using STRUCTURE software [39] and based on multivariate methods by using Discriminant Analysis of Principal Components (DAPC) [40] in the Adegenet software package in R [41]. Haplotypes were assigned to the clusters based on independent analyses of the SSR and MLST data. SSR inputs for the analysis were the amplicon sizes of the ten microsatellite markers (Table S1) from each isolate, whereas MLST inputs were each one of the polymorphic sites (SNPs and insertions/deletions) of the three genomic regions (Figure S1). Even though some polymorphic sites were consecutive and likely linked, each one of the 27 (as shown in results) was treated as a single locus (Figure S1). The three MLST genomic regions were aligned by using BioEdit 7.2.4 [38]; concatenated (total length of 1,244 bp), and then combined into unique haplotypes (Table S1A). Isolates containing identical haplotypes were considered as one, *i*.*e*., the dataset was clone-corrected.

A provisional genetic cluster assignment independent of geographical location was determined by using the Bayesian clustering algorithm implemented in the software STRUCTURE [39]. We used the admixture, correlated frequency model, and tested *K* (number of genetic clusters) = 1 to 10. Parameters were estimated under the null hypothesis of panmixia where loci are at Hardy-Weinberg (H-W) equilibrium. Twenty independent runs were performed with a burn-in period and run length of 50,000 and 500,000 iterations, respectively. The optimal number of populations (*K*) was estimated by using Structure Harvester [42] according to the *ad hoc* statistic Δ*K*, which is based on the rate of change in the log probability of the data between succeeding *K* values [43]. The assignment of each isolate/haplotype to a cluster was based on the quality threshold of *q*, which denotes the admixture proportion for each individual belonging to a cluster. In a situation where cluster *x* had the highest *q*-value for a determined individual, this individual was assigned to that cluster only if cluster *x* had a *q*-value of ≥0.75, or if the *q*-value was ≥0.5 and twice the *q*-value of another cluster. Clones whose *q-*values did not fit these parameters were not assigned to any specific cluster.

Violations of the assumptions in STRUCTURE (panmixia, H–W equilibrium, linkage equilibrium in clonal subgroups) can produce incorrect assignments; therefore, the results from STRUCTURE were compared to a second genetic cluster assignment by using DAPC. DAPC optimizes variation among clusters to the detriment of variation within clusters and, contrary to the results from STRUCTURE, is neutral to any *a priori* genetic hypothesis. The differentiation of individuals within and among populations was calculated by using Analysis of Molecular Variance (AMOVA) [44], which is based on a fixation index (*F*_ST_), using the Arlequin software package [45]. The resulting *F*_ST_ values were entered into a table and used to determine the most reliable genetic cluster model assignment for *G. morbida*.

Host specificity was also analyzed independently with both SSR and MLST data by using AMOVA. The analysis was only conducted for host species from which more than five *G. morbida*-isolates had been collected. *F*_ST_ *P*-values (α = 0.05) were used to determine host specificity.

#### Phylogenetic analyses, sexual reproduction/recombination, neutrality, and linkage disequilibrium tests

Due to the higher robustness of genetic clustering with the MLST data (see Results) and because more isolates were genotyped by using this approach, we performed most of the population genetic analyses with a focus on the MLST data.

**Figure 2:**
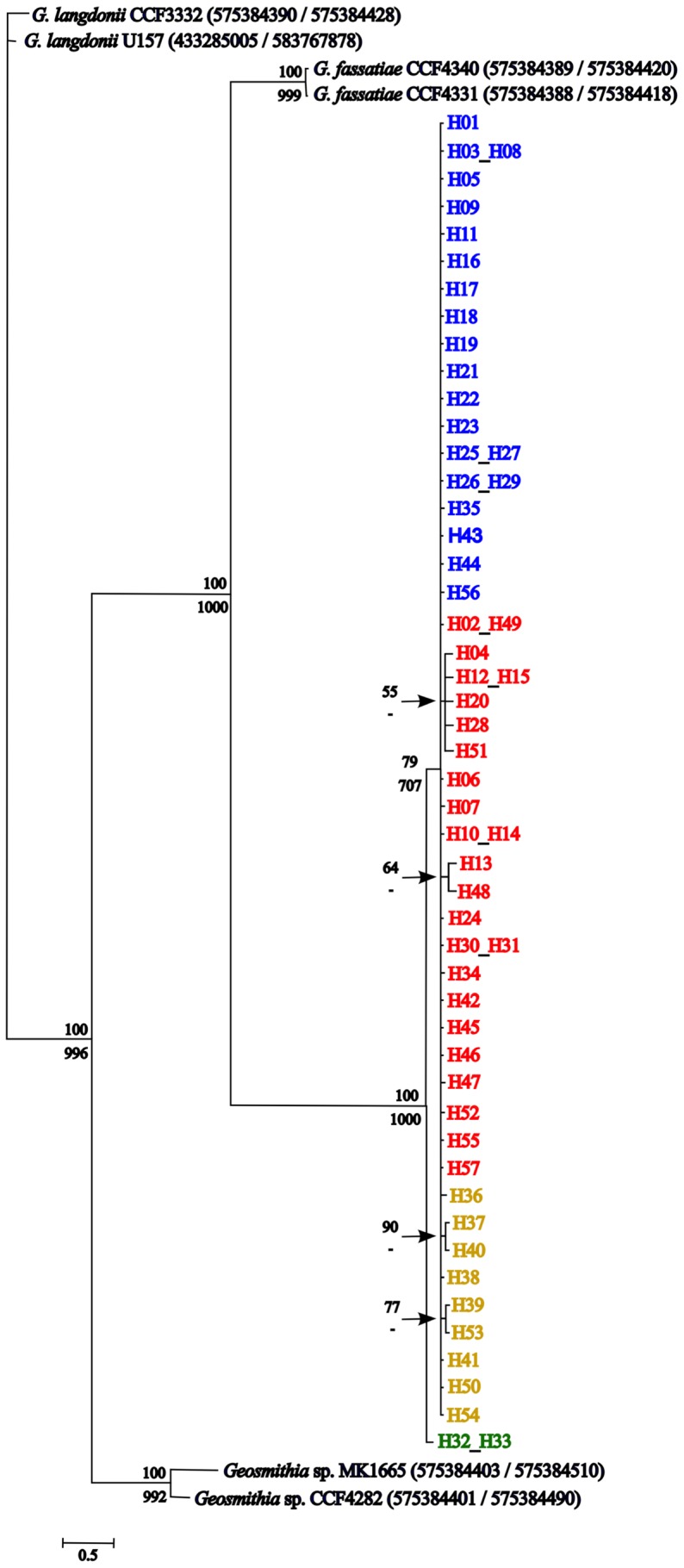
Unrooted phylogenetic tree of *Geosmithia* species based on ITS/BT sequences. A Bayesian analysis was performed for 1,500,000 generations by using a GTR-gamma distributed model of evolution (invariant sites). Bayesian percentages (≥50%) are depicted above each branch, and maximum likelihood bootstrap values (≥500) obtained by using PhyML (default parameters) are shown below most branches. *Geosmithia morbida* haplotypes are color coded according to their genetic cluster assignment (four-cluster-MLST-DAPC model, as in Figure 3) and haplotypes sharing the same ITS and BT sequences are co-located. Leaves pertaining to the same branch were arranged together according to their cluster assignment. GenBank accession numbers of other *Geosmithia* spp. are identified within parenthesis.

Phylogenetic analysis showing the relatedness of *G. morbida* haplotypes and other *Geosmithia* species was conducted by using two concatenated genomic regions (ITS/BT). MAP was not included because of the absence of sequence data for other species in public databases. Fifty-five sequences were compared, six of which corresponded to other species of *Geosmithia* downloaded from GenBank (accessions are shown in Figure 2); the others corresponded to *G. morbida* haplotypes. Because MAP was not considered, some of the haplotypes sharing the same ITS and BT sequences were combined in a single leaf (*e.g.*, H32 and H33, Figure 2). Sequences were aligned by using ClustalW [46] and trimmed in MEGA 5.05 [47], with lengths of 520 bp and 406 bp for ITS and BT, respectively. We performed two analyses: a Bayesian inference of phylogeny with a variant of Markov chain Monte Carlo [48] in MrBayes v3.1.2 [49] by using a general time-reversible model with inverse-gamma rates of evolution for 1,500,000 generations and a burn-in of 0.25; and a maximum-likelihood analysis in PhyML 3.0 [50] (http://www.atgc-montpellier.fr/phyml/) with default values except for bootstrap of 1,000 replicates. The unrooted phylogenetic tree obtained in MrBayes was formatted in MEGA5 [47] and branches with bootstrap values equal or greater than 50% were shown. Bootstrap values greater than 500 obtained in maximum-likelihood analysis were also indicated in that tree.

The program GenClone 2.0 [51] was used to calculate *p*_gen_, *i.e.*, the likelihood that MLST haplotypes resulted from sexual reproduction. Neutrality of the polymorphic loci was tested based on coalescent simulations with the program DnaSP 5.10 [52]; 1,000 replicates were used to test Tajima’s *D*; Fu and Li’s *D*, D*, *F* and F*; and Fu’s *F* neutrality statistics. MultiLocus 1.2 [53] was used to test linkage disequilibrium of alleles at different loci, and to test the null hypothesis of recombination. Indexes of association I_A_ and r-bar d were obtained with their corresponding probabilities by using 10,000 randomizations.

#### Private alleles

We used the most robust cluster assignment (four-cluster-MLST-DAPC model, as explained under “Genetic Structure”) to investigate private alleles in SSR analysis, *i.e.*, alleles found in only one cluster or one group of interest. This analysis was performed manually.

#### Geographical structure

Genotype (or haplotype) diversity of each geographic region was also calculated by using DnaSP 5.10 [52], measuring the genetic variance of haplotypes located in the same region. Significance of the proposed geographic structures was subsequently tested with Hudson’s Permtest, which computes the mean number of pairwise differences within (K*_ii_ or K*_jj_) and between groups (K*_ij_) along with the probability that K*_ij_ exceeds K*_ii_ by chance [54] (http://wwwabi.snv.jussieu.fr/public/mpweb/). Robustness of geographic clusters was calculated by using AMOVA in the Arlequin software package [44], [45].

## Results

### Isolation and identification of SSR sequences

Ten microsatellite (SSR) loci revealed a total of 59 alleles in *G. morbida* (Table S1). Amplicon sizes were compared, and two isolates (1348 and 1352) were identified as clonal because all but one amplicon from these isolates had the same size. Loci 2514 and 3416 failed to amplify in an unusually high percentage of samples (75% and 63%, respectively), from which other loci amplified consistently, which may indicate the presence of null alleles at those loci (Table S2).

### Isolation and identification of MLST sequences

From the set of 16 pairs of primers tested with *G. morbida* DNA as template (Table 2) all eight based on the *Fusarium solani* genome [34] failed to amplify a single well-defined band. The six pairs of primers designed based on the *G. morbida* genome produced a single amplicon (Table 2), and one pair corresponded to a polymorphic region (MAP) when the sequences of eight experimental isolates were compared (Table 1, isolates with two asterisks). ITS and BT produced polymorphic sequences [55], as reported in other species of *Geosmithia* [56], and were therefore used in this population genetic study. BT was originally amplified by using a pair of primers based on *Fusarium* spp. genome [36], but after sequencing the corresponding fragment from *G. morbida* one primer was refined accordingly (Table 2). We did not detect sequences with consistent noise or base call overlap, which would be an indication of different variants (alleles) of multi-copy genes (or genomic regions) within the same individual.

### Haplotype assignment

**Figure 3:**
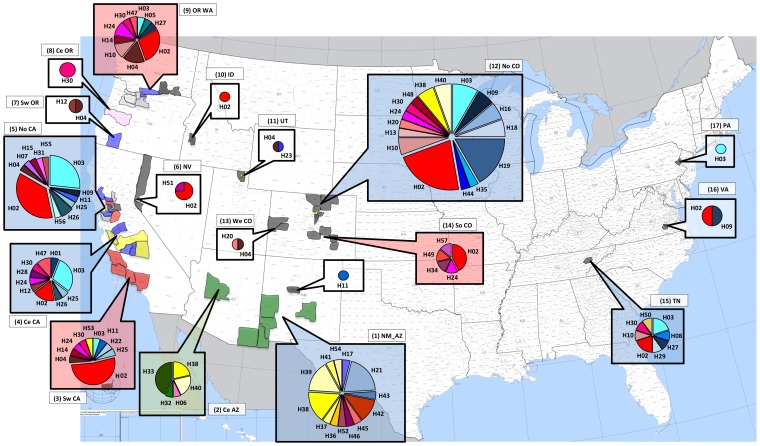
Distribution of 57 MLST-based *Geosmithia morbida* haplotypes in the United States. The size of wedges in each pie chart is proportional to the number of isolates. Haplotype colors relate to genetic clusters identified in the four-cluster-MLST-DAPC model where cluster 1 = shades of blue, cluster 2 = shades of red/brown, cluster 3 = shades of yellow, and cluster 4 = shades of green. Callouts are color-coded according to the three-region geographic-Hudson’s Permtest model, where: 1) blue = NM_AZ, central CA, northern CA, northern CO, and TN; 2) green = central AZ; and 3) red = southwestern CA, OR_WA, and southern CO. Callouts in white indicate regions not assessed by using Hudson’s Permtest. Counties are color-coded as in Figure 1B. (U.S. map adapted from US Census Bureau at https://www.census.gov/).

Using MLST data, 197 *G. morbida* isolates were placed into one of 57 haplotypes based on 11, 16, and 4 variants of ITS, BT, and MAP, respectively (Table S2 and Table 1), according to the combination of 7, 13, and 7 polymorphic loci, respectively (Figure S1). Multiple haplotypes were identified from isolates collected from different cankers on the same tree in cases from AZ, CA, CO, NM, TN, and VA (Table 1). Twenty-four of the haplotypes (42%) were represented by more than one isolate (Table 1). Overall, haplotypes H02 and H03 occurred the most frequently in the survey, representing 36% of all *G. morbida* isolates examined (Figure 3). They were detected in all geographic regions where more than 4 isolates were collected, except neither haplotype occurred in the native range of *J. major* (central AZ and NM_AZ), and haplotype H03 did not occur in southern CO (Figure 3). Haplotype H02 was especially abundant in the three geographic regions in CA (southwestern, central, and northern). Haplotype H03 was also abundant in northern CA and central CA but less so in the native range of *J. californica* in southwestern CA. Most of the geographic regions shared haplotypes with other regions, with the exception of central AZ and NM_AZ. In fact, most of the haplotypes present in those two regions were either exclusive or only shared between them, and all isolates from those locations were collected from *J. major* (Figure 3). The exceptions were haplotypes H38 and H40, which were also collected from a single county (Jefferson County) in northern CO, but from *J. nigra*. Isolate 1503 (Table 1) was not grouped with the other NM_AZ isolates because it was collected from *J. nigra* in an urban planting in Albuquerque (NM) and carried a haplotype that was not found in the native range of *J. major*.

The SSR data was not organized into haplotypes because no repeated haplotypes were identified when the allele sizes of the ten loci were analyzed, with the exception of isolates 1348 and 1352, as described above.

### Data analyses

#### Genetic structure

According to analysis with STRUCTURE, 107 *G. morbida* isolates were best organized into two clusters based on SSR allele frequency with a smaller Δ*K*-peak at 4 (Figure S2). Similarly, the clone-corrected MLST data from 57 haplotypes resulted in a Δ*K* peak at 4 following analysis with STRUCTURE (Figure S2).

**Figure 4:**
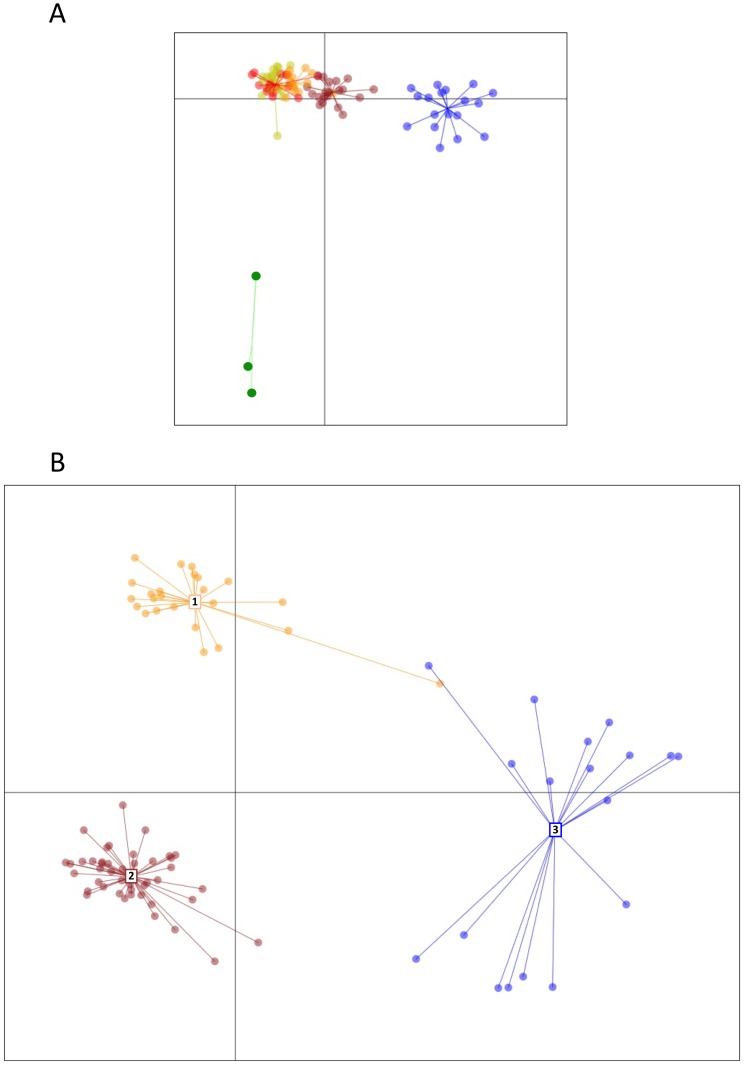
Coordinates of the SSR profile of 112 isolates of *G. morbida* based on DAPC analysis: of a theoretical *K* = 6 (A); and the same, but excluding the distant isolates in green, which resulted in *K* = 3 (B).

Assignment of the SSR data to genetic clusters by using DAPC resulted in an optimal theoretical *K* = 6 (Figure 4A). One cluster containing only three isolates, 1234, 1303, and 1307 (in green in Figure 4A) segregated from a group of five clusters. When the three distant isolates were removed and data re-analyzed, the remaining isolates were re-organized into three clusters (Figure 4B). Therefore, in further SSR-DAPC analysis we considered a *K* = 4, comprising the one cluster containing the three distant isolates (in green in Figure 4A) as well as the three additional clusters in Figure 4B. The fixation index of *K* = 4 was similar to the six-clusters-SSR-DAPC model obtained originally (Table 3).

**Table 3:** *Geosmithia morbida* molecular variance determined by AMOVA of Bayesian (Structure), DAPC, and Hudson’s Permtest analyses.

Number of clusters	Fixation indices (*F*_ST_)	Variation within/among population (%)
Genetic clusters (data/test)		
4 (MLST/Bayesian)	0.515	48.51/51.49
2 (MLST/Bayesian)	0.253	74.73/25.27
6 (SSR/DAPC)	0.425	57.50/42.50
4 (SSR/DAPC)	0.461	46.08/53.92
2 (MLST/DAPC)	0.739	26.13/73.87
4 (MLST/DAPC)	0.612	38.81/61.19
Geographic clusters		
17 regions	0.167	83.31/16.69
8 regions*	0.198	80.16/19.84
6 regions**	0.212	78.78/21.22
3 regions***	0.248	75.23/24.77

* 1) central AZ; 2) NM_AZ; 3) northern and central CA; 4) southwestern CA; 5) OR_WA; 6) northern CO; 7) southern CO; and 8) TN.

** 1) central AZ; 2) NM_AZ; 3) northern and central CA and northern CO; 4) southwestern CA; 5) OR_WA and southern CO; and 6) TN.

*** The three “macro” regions were: 1) NM_AZ, central CA, northern CA, northern CO, and TN; 2) central AZ and 3) southwestern CA, OR_WA, and southern CO.

**Figure 5:**
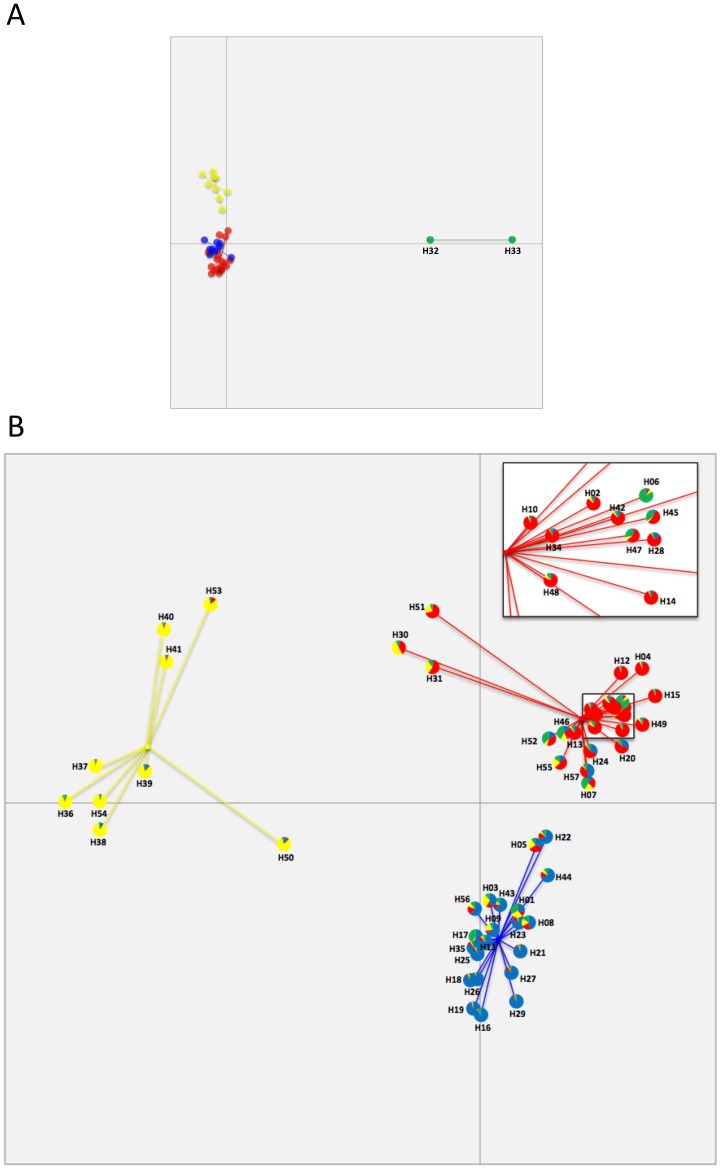
Coordinates of 57 (A) and 55 (B) haplotypes of *Geosmithia morbida* from the MLST-DAPC model. The most distant cluster (cluster 4 in green) comprised of haplotypes H32 and H33 is identified **(A)**, as well as the coordinates of all remaining haplotypes when H32 and H33 were excluded **(B)**. A comparison between the assignments of the MLST-DAPC and MLST-STRUCTURE models are shown in detail. Pie charts give the probability of assignment of haplotypes to the four genetic clusters obtained in the four-clusters-MLST-STRUCTURE model. They are represented by colors, cluster 1 = blue, cluster 2 = red, cluster 3 = yellow and cluster 4 = green. Haplotypes in the box (in **B**) were amplified for better resolution.

The MLST data analyzed with DAPC was first assigned to two clusters containing 55 and 2 haplotypes each (Figure 5A). When the distant cluster containing haplotypes H32 and H33 was excluded (in green in Figure 5A), the remaining 55 haplotypes segregated into three distinct clusters (Figure 5B). Fixation indices were similar for both two and four MLST-DAPC-clusters (Table 3). When the cluster assignment of the haplotypes on the four-cluster-MLST-STRUCTURE model was compared to the four-cluster-MLST-DAPC, they were mostly correlated, despite some discrepancies (Figure 5A, green cluster; and Figure 5B, all clusters). The four-cluster-MLST-DAPC model had a high (>0.25) fixation index and it was the most informative when compared to other models of genetic and geographic (as described below) clustering of *G. morbida* isolates. Therefore, the four-cluster-MLST-DAPC model was used to assign haplotypes to clusters in further analyses (Table 1 and Figure 3).

Examining the haplotype assignment, based on the four-cluster-MLST-DAPC model, cluster 1 (blue color in pie charts of Figure 3) contained 21 haplotypes (63 isolates) including the abundant and widely distributed haplotype H03 (Table 1 and Figure 3). Cluster 1 was especially abundant in northern CO, northern CA, central CA and TN. Cluster 2 (red in pie charts, Figure 3) contained 25 haplotypes (103 isolates), including the abundant and widely distributed haplotype H02. Cluster 2 was especially abundant in the three regions in CA, OR_WA, and in southern CO, where it accounted for more than half of the individuals (Table 1 and Figure 3). Cluster 3 (yellow/amber in pie charts, Figure 3) was represented by 9 haplotypes (24 isolates) and was abundant in central AZ and NM_AZ, but not present in most of the other regions, except for two haplotypes in northern CO and one in southwestern CA and TN (Table 1 and Figure 3). Cluster 4 (green in pies charts, Figure 3) contained 2 haplotypes (7 isolates) that were only detected from *J. major* in central AZ (Table 1 and Figure 3).

We found correspondence in cluster assignment between MLST and SSR data by using DAPC (Figure 4A, 4B, 5A, and 5B). The outliers (isolates) 1234, 1303, and 1307 in SSR analysis (Figure 4A) corresponded to H33 (Figure 5A), which is also one of the outliers in MLST analysis. The single isolate (1299) pertaining to H32 (Figure 5A) was not assessed by the SSR method. Most of the isolates (75%) in SSR-cluster 1 corresponded to cluster 2 in MLST analysis, and most of the isolates (47%) in SSR-cluster 3 corresponded to cluster 3 in MLST analysis. The SSR-cluster 2 was comprised of 67% of MLST-cluster 2 and 33% of MLST-cluster 1 isolates.

#### Host specificity

Based on pairwise *F*_ST_ analysis, the SSR and MLST data indicated that *J. major* isolates were different from *J. californica*, *J. hindsii*, *J. nigra* and *J. regia* isolates collectively, whereas *J. nigra* isolates were different from *J. californica* isolates (Table S3).

#### Phylogenetic analyses

In a phylogenetic tree based on Bayesian inference with ITS and BT sequences (Figure 2), all 57 haplotypes of *G. morbida* (Table S2) grouped in the same clade with strong bootstrap support, apart from several other *Geosmithia* species. Within the *G*. *morbida* clade, isolates in the haplotypes H32 and H33 formed their own sub-clade with strong bootstrap support and the remaining haplotypes clustered together into a second sub-clade.

#### Sexual reproduction/recombination, neutrality, and linkage disequilibrium tests

Independent analyses of both the whole set of 57 haplotypes and haplotypes assigned to MLST-DAPC clusters showed that the likelihood of the observed haplotypes arising from sexual reproduction was extremely low (*p*_gen_ <0.05). In both the complete dataset and clone-corrected data, the *r*_d_ test rejected the null hypothesis of recombination in MLST loci (*P*<0.001) and we obtained similar results by using SSR data (*P*<0.001). The polymorphic MLST-loci were all selectively neutral, whereas polymorphisms in SSR loci were presumed neutral as they were extra-genic. Overall, the three genomic regions used in the MLST analysis were in linkage disequilibrium (index of association = 0.8005, *P*<0.001) and pairwise tests indicated the same (index of association = 0.8005, *P* = 0.029).

#### Private alleles

Private alleles may indicate restrictions in dispersal or evolutionary isolation. The four-cluster-MLST-DAPC model was used to investigate private alleles in SSR analysis (Table S2). In this regard, isolates in cluster 4, showed a high number of private alleles and included private alleles at seven of ten loci examined. Moreover, the most common allele at locus 194 (270 bp), locus 2849 (310 bp and 316), and locus 4045 (184 bp) were completely absent from isolates of cluster 4, reinforcing their genetic distinctiveness. Isolates belonging to cluster 3 showed private alleles at SSR loci 223, 194, 1851, and 6823; cluster 2 had private alleles at 2849, 3416 and 7713, and cluster 1 at 4045, 6823 and 7713 (Table S2).

#### Geographical structure

*Geosmithia morbida* isolates were placed initially into one of 17 geographic regions based on the proximity of the counties where the isolates were collected. These regions, while arbitrarily determined, were for the most part spatially isolated from one another (Figure 1B). Even though the fixation index (*F*_ST_ = 0.167) (Table 3) was not low when the 17 regions were tested, the population structure in some regions was not different from others according to Hudson’s Permtest [54]. Because of that, a series of Hudson’s test-based pairwise comparisons of 8 and then 6 regions (regions that we compared are described in Table 3) that included more than four isolates indicated that the populations could be combined into three different (*P*<0.001) macro regions encompassing: 1) NM_AZ, central CA, northern CA, northern CO, and TN; 2) central AZ and 3) southwestern CA, OR_WA, and southern CO (depicted in callouts in Figure 3). Therefore the three-region geographic model was the most robust when compared to other geographic models. This conclusion is based on fixation indices (*F*_ST_) that identified the lowest within and highest among population variation (Table 3), and all pairwise *F*_ST_ values were significant (Table 4). The haplotype diversity was high across all three macro regions [0.70 (SD±0.02)]. The values obtained for the three regions individually were 0.67 (SD±0.03), 0.77 (SD±0.08), and 0.54 (SD±0.08) in: 1) NM_AZ/Ce CA/No CA/No CO/TN; 2) Ce AZ; and 3) Sw CA/OR_WA/So CO, respectively.

**Table 4:** Pairwise *F*_ST_ values calculated for the three-population geographical model observed with Hudson’s Permtest.

Geographic cluster	1	2	3
1	–	0.423*	0.109*
2	0.423*	–	0.491*

Significant (*P*<0.05) values are denoted by (*).

Geographical regions: 1) NM_AZ/Ce CA/No CA/No CO/TN; 2) Ce AZ; and 3) Sw CA/OR_WA/So CO.

## Discussion

### Isolates and genetic diversity

*Geosmithia morbida* was isolated consistently from cankers surrounding WTB galleries from all *Juglans* and *Pterocarya* showing TCD symptoms and in all regions that we surveyed (Fig. 1A), with the exception of *J. major* in Texas where the WTB was not detected [23]. Considering the substrate specificity of bark beetles and *Geosmithia* associates to their respective plant hosts [56], and the highly diverse and complex genetic structure in *G. morbida* that we and others [10], [26], [27] have observed, it is unlikely that the current TCD epidemic was a result of a new association between the WTB and *G. morbida*.

The number of polymorphic MLST loci described here and elsewhere [10] was not low, as we had first expected. However, it is unlikely that haplotypes are admixed (sexual recombinants) because no teleomorph has been observed and according to tests for sexual reproduction, neutrality, and disequilibrium, sexual recombination in *G. morbida* is nonexistent or infrequent. Considerable intra-species variation of the ITS rDNA region has been reported in other *Geosmithia* species [10], [56], and this may be a common characteristic of this genus. Thus, this variability does not necessarily support the hypothesis that *G. morbida* is a species complex.

All isolates were identified initially as *G. morbida* based on the similarity of their morphological characteristics and their growth patterns and color in culture. However, the placement of haplotypes H32 and H33 in a group distinct from the other *G. morbida* haplotypes according to the ITS/BT-based phylogenetic tree and based on analyses of genetic and geographic cluster assignments could be viewed as evidence for a hypothetical cryptic species (Figure 2, 4A, and 5A). All isolates characterized by H32 and H33 were derived from *J. major*, whereas the other haplotypes were collected from many *Juglans* and *Pterocarya* species. However, in previous work, our team has found that isolate 1234 (characterized by H33 and first documented from *J. major* in Kolařík et al. 2011 [11]) is indistinguishable from other *G. morbida* isolates in terms of its morphology and pathogenicity. A more thorough multi-gene phylogenetic analysis in the future including other species of *Geosmithia* and more isolates of *G. morbida* from *J. major* in the southwestern USA and Mexico might be necessary to investigate the potential for cryptic species related to *G. morbida*.

### Genetic, geographic, and host groupings

Our results indicated that the *G. morbida* genetic structure in the USA was best explained by four distinct genetic groups. Cluster assignment correspondence between MLST and SSR by using the DAPC method was moderate, probably due to some missing data in SSR (Table S2). The most robust analysis was achieved by DAPC analysis with MLST data (Figure 5 A, B and Table 3), with support of STRUCTURE. The four-cluster-MLST-DAPC model differed from the two clusters identified by Hadziabdic and collaborators [27], probably because we sampled more extensively and in areas not sampled in their study.

Haplotypes in genetic clusters 1 and 2 (blue and red) dominated in locations outside of AZ and NM (*i.e*., outside the native range of *J. major*); vice versa for clusters 3 and 4 (yellow and green) (Figure 3). The high proportion of widely-dispersed, closely-related *G. morbida* haplotypes supports the hypothesis of a recent invasion by at least three genetic groups into regions other than central AZ and NM_AZ (Figure 3).

The haplotypes were further grouped into three diverse geographic groups (callouts in Figure 3), possibly indicating that regions within the same group exchanged or had a common source of haplotypes. A comparable genetic and geographic structure was observed for *Ophiognomonia clavigignenti-juglandacearum*, an exotic canker pathogen of *J. cinerea* in North America [57], which consisted of three genetic clusters of haplotypes that were geographically disjunct. In the past, many epidemics caused by invasive plants, pathogens, or insects were assumed to be initiated by a single introduction event followed by a radiation from the introduction point. However, several recent studies have found that this is likely not the case and that, in most invasion events, multiple introductions have occurred followed by migration and admixture of populations [58]–[61]. This may be particularly true for forest pathogens as damage caused by these organisms may go unnoticed and undetected for decades in more remote areas. This allows greater migration and genetic drift to occur between and among populations. Based on the amount of genetic diversity observed in the ITS, BT, and MAP regions and the fact that *G. morbida* is present but does not cause mortality in some *Juglans* species, it is likely that this fungus was present in at least one location in North America for decades, being moved virtually unnoticed amongst *Juglans* populations by the WTB. However, once mortality on *J. nigra* was observed out of its natural range and the fungus was detected and described for the first time, the full extent of the distribution of *G. morbida* was finally realized [4], [9], [11].

Although we found some evidence of host specialization, these data were strongly influenced by the uneven geographic distribution of the *Juglans* species, *i.e.*, host and region were highly confounded. *Geosmithia morbida* isolates from *J. californica* and *J. major* were derived almost exclusively from trees in or near their native ranges, whereas isolates from other hosts were collected in multiple regions and from planted trees. Isolates from the Eurasian native, *J. regia*, were derived primarily from adventive orchards in CA and two outlying samples from CO and UT. Moreover, the number of isolates collected from the hosts was extremely unbalanced. Further studies that extend the work of Utley *et al*. [62] by evaluating virulence of selected isolates on various hosts are needed to confirm any evidence of host specialization.

### Introduction of multiple *G. morbida* haplotypes to geographically isolated areas

We recovered multiple *G. morbida* haplotypes representing different genetic clusters from different cankers on the same tree; their introduction could have occurred in different events or all at once from a single WTB-infested log. Even small infested logs harbor large numbers of beetles [63]. The WTB is not considered a strong flier [25] and even though *G. morbida* produces dry conidia, they are unlikely to be dispersed by the wind because the spores are only formed in beetle galleries and feeding sites [62]. Therefore, the most probable means of *G. morbida* introduction into geographically isolated areas is by importation of contaminated logs or wood products (with bark attached) [25]. The anthropogenic movement of wood containing many haplotypes would explain the complex mixture of *G. morbida* haplotypes observed at most locations.

The high proportions of haplotypes H02 and H03 in most of the regions may represent their relative abundance in the original or secondary invasive population; the frequency of haplotype reintroduction during subsequent dispersal events; or it could indicate some type of competitive advantage [26], [55].

### Possible origin of *G. morbida* isolates in the TCD epidemic: *J. major* native range as primary source

We hypothesized that *G. morbida* was dispersed from the native range of *J. major*, where the WTB vector was first collected [7], [21]. There is circumstantial evidence of an isolated introduction of *G. morbida* (H38 and H40) from central AZ into northern CO (yellow in pie charts in Figure 3), but these haplotypes represent a small fraction of the total number. Thus, the two haplotypes were probably part of an independent and secondary introduction of *G. morbida* into northern CO.

In general, we did not detect haplotypes collected from *J. major* in AZ and NM in other areas in the USA. Although we did not find shared haplotypes, the Hudson’s Permtest [54] analysis indicated that the genetic makeup of haplotypes from NM_AZ was not significantly different from those found in central CA, northern CA, northern CO, and TN (blue callouts in Figure 3). The high number of exclusive *G. morbida* haplotypes collected from *J. major* in central AZ and NM_AZ (Figure 3) and the large number of private alleles, combined with the absence in this region of the two most frequently occurring haplotypes (H02 and H03), make it unlikely that these two regions are the direct geographic sources of *G. morbida* isolates associated with TCD outbreaks in the majority of locations. *Juglans major* has an extensive but non-contiguous range that extends south into Mexico [64], and our sampling was restricted to the extreme northern portion of the full range (Figure 1A) of this species. If *G. morbida* is present throughout the range of *J. major*, its population structure may be variable because of genetic isolation. Even within central AZ and NM_AZ, we noted what appeared to be geographically isolated genetic clusters of *G. morbida* (Table 1). A more intensive survey in unsampled regions of *J. major* is warranted.

### Possible origin of *G. morbida* isolates in the TCD epidemic: *J. californica* native range as primary source

Another possible source of *G. morbida* haplotypes causing the TCD epidemic could be southwestern CA. All *G. morbida* isolates from this region were collected from *J. californica*, a species that may be a native host of the WTB and its symbiont *G. morbida* [7]. Moreover, a very high proportion (89%) of the haplotypes identified in southwestern CA, in particular haplotypes H02 and H03, was also found in at least one other region (Figure 3). Furthermore, the result of the geographic structure analysis indicated that southwestern CA was similar to OR_WA and southern CO (Figure 3).

Geographic structure analysis also indicated genetic similarities among *G. morbida* haplotypes in northern CO, TN, central CA and northern CA (Figure 3), with the latter region representing the natural range of *J. hindsii* (Figure 1A). Central CA and northern CA shared 88% and 83% of their haplotypes with other regions (Figure 3). It is possible that the TCD epidemic was triggered when infested raw walnut wood products were moved from northern CA to northern CO, or vice versa.

### *Geosmithia morbida* dispersal

Regardless of its origin, TCD was first noted after its appearance on the highly susceptible *J. nigra* [4], [10], probably as a consequence of transportation of infested logs that allowed movement of WTB and *G. morbida* into regions that they would not have reached naturally. Introduction to new sites could also have been facilitated by tourists/campers carrying even small amounts of wood. For example, there were more than 13 million visitors to Arizona’s State and National Parks from 2001–2012 (www.azot.gov). Visitor attendance during this period to California’s State Parks alone was even higher, ranging from 85.5 million (2001–2002) to 67.9 million (2011–2012) (http://www.parks.ca.gov/?page_id=23308). The genetic makeup of the *G. morbida* populations in regions where *J. nigra* has been affected suggests that the outbreaks in most of the USA are related to those in CA and from a fungus population that was already established in at least one species of *Juglans*.

## Conclusion

This study helped to explain the genetic structure of *G. morbida* and its multi-factorial and disjunct distribution in the USA. Even though the observation of TCD in *Juglans* and *Pterocarya* species has only been recent, the observed haplotype diversity and the genetic complexity of *G. morbida* indicate it has been in association with at least one *Juglans* spp. and the WTB for a long period of time. Some haplotypes and genetic clusters were found in specific regions and in association only with certain *Juglans* species. The scattered geographic distribution of genetic clusters indicates that *G. morbida* was disseminated several times and from several sources and most likely by transportation in beetle-infested wood.

## Supporting Information

Figure S1 **Twenty-seven nucleotide polymorphic loci detected in three genomic regions in** ***G. morbida*** **.** Sequencing ITS, BT and MAP of *G. morbida* isolates resulted in 7, 13 and 7 polymorphisms, respectively. Those were combined in 57 haplotypes (Table S2). Polymorphisms are depicted in colors and loci are identified.
(PDF)

Figure S2 **Results of Δ** ***K*** **computation for** ***Geosmithia morbida*** **isolates.** Scenario where *K* = 1-10 (20 iterations) by using STRUCTURE [39]. SSR data supports clusters of 2 and 4, whereas MLST data supports a cluster of 4.
(TIF)

Table S1 **Microsatellite (SSR) loci used to characterize** ***Geosmithia morbida*** **isolates**.
(PDF)

Table S2 **Collection Information of 209** ***Geosmithia morbida*** **-isolates and their MLST and SSR profile.**
(XLSX)

Table S3 **Host specificity analysis.** *F*_ST_ values calculated using both MLST (above diagonal) and SSR (below diagonal) data, grouping isolates by host. Significant (*P*<0.05) *F*_ST_ values are denoted by (*).
(XLSX)

File S1 **Development of MLST-primers based on partial sequencing of the** ***Geosmithia morbida*** **(CBS 124663) genome.**
(DOCX)
