# Supplementary material for: Population Structure of Geosmithia morbida, the Causal Agent of Thousand Cankers Disease of Walnut Trees in the United States
Source: PLoS One. 2014 Nov 13;9(11):e112847. doi: 10.1371/journal.pone.0112847 (PMC4231075; doi:10.1371/journal.pone.0112847)
Supplement: Figure S1 — Twenty-seven nucleotide polymorphic loci detected in three genomic regions in G. morbida . Sequencing ITS, BT and MAP of G. morbida isolates resulted in 7, 13 and 7 polymorphisms, respectively. Those were combined in 57 haplotypes (Table S2). Polymorphisms are depicted in colors and loci are identified. (PDF) [file pone.0112847.s001.pdf]

ITS

```
Group09      CAGTTGCTTCGGCGGCCCCGCCCCGGGCGCCTCGGGACCCCCCCCC-TAGGGGG-CCCCG 58
Group06      CAGTTGCTTCGGCGGCCCCGCCCCGGGCGCCTCGGGATCCCCCCCCCTAGGGGG-CCCCG 59
Group01      CAGTTGCTTCGGCGGCCCCGCCCCGGGCGCCTCGGGATCCCCCCCC-TAGGGGG-CCCCG 58
Group07      CAGTTGCTTCGGCGGCCCCGCCCCGGGCGCCTCGGGATCCCCCCCCTTAGGGGG-CCCCG 59
Group04      CAGTTGCTTCGGCGGCCCCGCCCCGGGCGCCTCGGGATC--CCCCC-TAGGGGG-CCCCG 56
Group10      CAGTTGCTTCGGCGGCCCCGCCCCGGGCGCCTCGGGATCCCCCCCC-TAGGGGGGCCCCG 59
Group08      CAGTTGCTTCGGCGGCCCCGCCCCGGGCGCCTCGGGATCTCCCCCC-TAGGGGGGCCCCG 59
Group02      CAGTTGCTTCGGCGGCCCCGCCCCGGGCGCCTCGGGATCTCCCCCC-TAGGGGG-CCCCG 58
Group03      CAGTTGCTTCGGCGGCCCCGCCCCGGGCGCCTCGGGATC--CCCCC-TAGGGGG-CCCCG 56
Group05      CAGTTGCTTCGGCGGCCCCGCCCCGGGCGCCTCGGGATC-CCCCCC-TAGGGGGGCCCCG 58
Group11      CAGTTGCTTCGGCGGCCCCGCCCCGGGCGCCTCGGGATCCCCCCCC-TAGGGGG-CCCCG 58
Loci         *************************************1*23*****4*******5*****

Group09      CGGGCGACCCGGACCCAGGCGACCGCCGGGGGACCCAAACTCTT-GTCTTTTAGTGTATT 117
Group06      CGGGCGACCCGGACCCAGGCGACCGCCGGGGGACCCAAACTCTT-GTCTTTTAGTGTATT 118
Group01      CGGGCGACCCGGACCCAGGCGACCGCCGGGGGACCCAAACTCTT-GTCTTTTAGTGTATT 117
Group07      CGGGCGACCCGGACCCAGGCGACCGCCGGGGGACCCAAACTCTT-GTCTTTTAGTGTATT 118
Group04      CGGGCGACCCGGACCCAGGCGACCGCCGGGGGACCCAAACTCTT-GTCTTTTAGTGTATT 115
Group10      CGGGCGACCCGGACCCAGGCGACCGCCGGGGGACCCAAACTCTT-GTCTTTTAGTGTATT 118
Group08      CGGGCGACCCGGACCCAGGCGACCGCCGGGGGACCCAAACTCTT-GTCTTTTAGTGTATT 118
Group02      CGGGCGACCCGGACCCAGGCGACCGCCGGGGGACCCAAACTCTT-GTCTTTTAGTGTATT 117
Group03      CGGGCGACCCGGACCCAGGCGACCGCCGGGGGACCCAAACTCTTTGTCTTTTAGTGTATT 116
Group05      CGGGCGACCCGGACCCAGGCGACCGCCGGGGGACCCAAACTCTT-GTCTTTTAGTGTATT 117
Group11      CGGGCGACCCGGACCCAGGCGACCGCCGGGGGACCCAAACTCTT-GTCTTTTAGTGTATT 117
Loci         ********************************************6***************

Group09      ATCTGAGTGGCATAAGCAAATAAATTAAAACTTTCAGCAACGGATCTCTTGGTTCTGGCA 177
Group06      ATCTGAGTGGCATAAGCAAATAAATTAAAACTTTCAGCAACGGATCTCTTGGTTCTGGCA 178
Group01      ATCTGAGTGGCATAAGCAAATAAATTAAAACTTTCAGCAACGGATCTCTTGGTTCTGGCA 177
Group07      ATCTGAGTGGCATAAGCAAATAAATTAAAACTTTCAGCAACGGATCTCTTGGTTCTGGCA 178
Group04      ATCTGAGTGGCATAAGCAAATAAATTAAAACTTTCAGCAACGGATCTCTTGGTTCTGGCA 175
Group10      ATCTGAGTGGCATAAGCAAATAAATTAAAACTTTCAGCAACGGATCTCTTGGTTCTGGCA 178
Group08      ATCTGAGTGGCATAAGCAAATAAATTAAAACTTTCAGCAACGGATCTCTTGGTTCTGGCA 178
Group02      ATCTGAGTGGCATAAGCAAATAAATTAAAACTTTCAGCAACGGATCTCTTGGTTCTGGCA 177
Group03      ATCTGAGTGGCATAAGCAAATAAATTAAAACTTTCAGCAACGGATCTCTTGGTTCTGGCA 176
Group05      ATCTGAGTGGCATAAGCAAATAAATTAAAACTTTCAGCAACGGATCTCTTGGTTCTGGCA 177
Group11      ATCTGAGTGGCATAAGCAAATAAATTAAAACTTTCAGCAACGGATCTCTTGGTTCTGGCA 177
             ************************************************************

Group09      TCGATGAAGAACGCAGCGAAATGCGATAAGTAATGTGAATTGCAGAATTCAGTGAATCAT 237
Group06      TCGATGAAGAACGCAGCGAAATGCGATAAGTAATGTGAATTGCAGAATTCAGTGAATCAT 238
Group01      TCGATGAAGAACGCAGCGAAATGCGATAAGTAATGTGAATTGCAGAATTCAGTGAATCAT 237
Group07      TCGATGAAGAACGCAGCGAAATGCGATAAGTAATGTGAATTGCAGAATTCAGTGAATCAT 238
Group04      TCGATGAAGAACGCAGCGAAATGCGATAAGTAATGTGAATTGCAGAATTCAGTGAATCAT 235
Group10      TCGATGAAGAACGCAGCGAAATGCGATAAGTAATGTGAATTGCAGAATTCAGTGAATCAT 238
Group08      TCGATGAAGAACGCAGCGAAATGCGATAAGTAATGTGAATTGCAGAATTCAGTGAATCAT 238
Group02      TCGATGAAGAACGCAGCGAAATGCGATAAGTAATGTGAATTGCAGAATTCAGTGAATCAT 237
Group03      TCGATGAAGAACGCAGCGAAATGCGATAAGTAATGTGAATTGCAGAATTCAGTGAATCAT 236
Group05      TCGATGAAGAACGCAGCGAAATGCGATAAGTAATGTGAATTGCAGAATTCAGTGAATCAT 237
Group11      TCGATGAAGAACGCAGCGAAATGCGATAAGTAATGTGAATTGCAGAATTCAGTGAATCAT 237
             ************************************************************

Group09      CGAATCTTTGAACGCACATTGCGCCCGCCAGTACTCTGGCGGGCATGCCTGTCCGAGCGT 297
Group06      CGAATCTTTGAACGCACATTGCGCCCGCCAGTACTCTGGCGGGCATGCCTGTCCGAGCGT 298
Group01      CGAATCTTTGAACGCACATTGCGCCCGCCAGTACTCTGGCGGGCATGCCTGTCCGAGCGT 297
Group07      CGAATCTTTGAACGCACATTGCGCCCGCCAGTACTCTGGCGGGCATGCCTGTCCGAGCGT 298
Group04      CGAATCTTTGAACGCACATTGCGCCCGCCAGTACTCTGGCGGGCATGCCTGTCCGAGCGT 295
Group10      CGAATCTTTGAACGCACATTGCGCCCGCCAGTACTCTGGCGGGCATGCCTGTCCGAGCGT 298
Group08      CGAATCTTTGAACGCACATTGCGCCCGCCAGTACTCTGGCGGGCATGCCTGTCCGAGCGT 298
Group02      CGAATCTTTGAACGCACATTGCGCCCGCCAGTACTCTGGCGGGCATGCCTGTCCGAGCGT 297
Group03      CGAATCTTTGAACGCACATTGCGCCCGCCAGTACTCTGGCGGGCATGCCTGTCCGAGCGT 296
Group05      CGAATCTTTGAACGCACATTGCGCCCGCCAGTACTCTGGCGGGCATGCCTGTCCGAGCGT 297
Group11      CGAATCTTTGAACGCACATTGCGCCCGCCAGTACTCTGGCGGGCATGCCTGTCCGAGCGT 297
             ************************************************************
```

```
Group09      CATTTCAACCCTCAGCACCCCCGTTCGCGGCGGGAGCTGGCGTTGGGGATCGGCCGTCCT 357
Group06      CATTTCAACCCTCAGCACCCCCGTTCGCGGCGGGAGCTGGCGTTGGGGATCGGCCGTCCT 358
Group01      CATTTCAACCCTCAGCACCCCCGTTCGCGGCGGGAGCTGGCGTTGGGGATCGGCCGTCCT 357
Group07      CATTTCAACCCTCAGCACCCCCGTTCGCGGCGGGAGCTGGCGTTGGGGATCGGCCGTCCT 358
Group04      CATTTCAACCCTCAGCACCCCCGTTCGCGGCGGGAGCTGGCGTTGGGGATCGGCCGTCCT 355
Group10      CATTTCAACCCTCAGCACCCCCGTTCGCGGCGGGAGCTGGCGTTGGGGATCGGCCGTCCT 358
Group08      CATTTCAACCCTCAGCACCCCCGTTCGCGGCGGGAGCTGGCGTTGGGGATCGGCCGTCCT 358
Group02      CATTTCAACCCTCAGCACCCCCGTTCGCGGCGGGAGCTGGCGTTGGGGATCGGCCGTCCT 357
Group03      CATTTCAACCCTCAGCACCCCCGTTCGCGGCGGGAGCTGGCGTTGGGGATCGGCCGTCCT 356
Group05      CATTTCAACCCTCAGCACCCCCGTTCGCGGCGGGAGCTGGCGTTGGGGATCGGCCGTCCT 357
Group11      CATTTCAACCCTCAGCACCCCCGTTCGCGGCGGGAGCTGGCGTTGGGGATCGGCCGTCCT 357
             ************************************************************

Group09      CTGCGGCGGCCGGCCCCGAAACGAAGTGGCGGTCACGTCGCGACCTCCTCTGCGTAGTAG 417
Group06      CTGCGGCGGCCGGCCCCGAAACGAAGTGGCGGTCACGTCGCGACCTCCTCTGCGTAGTAG 418
Group01      CTGCGGCGGCCGGCCCCGAAACGAAGTGGCGGTCACGTCGCGACCTCCTCTGCGTAGTAG 417
Group07      CTGCGGCGGCCGGCCCCGAAACGAAGTGGCGGTCACGTCGCGACCTCCTCTGCGTAGTAG 418
Group04      CTGCGGCGGCCGGCCCCGAAACGAAGTGGCGGTCACGTCGCGACCTCCTCTGCGTAGTAG 415
Group10      CTGCGGCGGCCGGCCCCGAAACGAAGTGGCGGTCACGTCGCGACCTCCTCTGCGTAGTAG 418
Group08      CTGCGGCGGCCGGCCCCGAAACGAAGTGGCGGTCACGTCGCGACCTCCTCTGCGTAGTAG 418
Group02      CTGCGGCGGCCGGCCCCGAAACGAAGTGGCGGTCACGTCGCGACCTCCTCTGCGTAGTAG 417
Group03      CTGCGGCGGCCGGCCCCGAAACGAAGTGGCGGTCACGTCGCGACCTCCTCTGCGTAGTAG 416
Group05      CTGCGGCGGCCGGCCCCGAAACGAAGTGGCGGTCACGTCGCGACCTCCTCTGCGTAGTAG 417
Group11      CTGCGGCGGCCGGCCCCGAAACGAAGTGGCGGTCACGTCGCGACCTCCTCTGCGTAGTAG 417
             ************************************************************

Group09      CAATATCTCGCAGCCTGGATAGCGGCGCGGCCACGCCGTAAAACCCCAACTATACCAAGG 477
Group06      CAATATCTCGCAGCCTGGATAGCGGCGCGGCCACGCCGTAAAACCCCAACTATACCAAGG 478
Group01      CAATATCTCGCAGCCTGGATAGCGGCGCGGCCACGCCGTAAAACCCCAACTATACCAAGG 477
Group07      CAATATCTCGCAGCCTGGATAGCGGCGCGGCCACGCCGTAAAACCCCAACTATACCAAGG 478
Group04      CAATATCTCGCAGCCTGGATAGCGGCGCGGCCACGCCGTAAAACCCCAACTATACCAAGG 475
Group10      CAATATCTCGCAGCCTGGATAGCGGCGCGGCCACGCCGTAAAACCCCAACTATACCAAGG 478
Group08      CAATATCTCGCAGCCTGGATAGCGGCGCGGCCACGCCGTAAAACCCCAACTATACCAAGG 478
Group02      CAATATCTCGCAGCCTGGATAGCGGCGCGGCCACGCCGTAAAACCCCAACTATACCAAGG 477
Group03      CAATATCTCGCAGCCTGGATAGCGGCGCGGCCACGCCGTAAAACCCCAACTATACCAAGG 476
Group05      CAATATCTCGCAGCCTGGATAGCGGCGCGGCCACGCCGTAAAACCCCAACTATACCAAGG 477
Group11      CAGTATCTCGCAGCCTGGATAGCGGCGCGGCCACGCCGTAAAACCCCAACTATACCAAGG 477
Loci         **7*********************************************************

Group09      TTGACCTCGGATCAGGTAGGAATACCCGCTGAACTTAA 515
Group06      TTGACCTCGGATCAGGTAGGAATACCCGCTGAACTTAA 516
Group01      TTGACCTCGGATCAGGTAGGAATACCCGCTGAACTTAA 515
Group07      TTGACCTCGGATCAGGTAGGAATACCCGCTGAACTTAA 516
Group04      TTGACCTCGGATCAGGTAGGAATACCCGCTGAACTTAA 513
Group10      TTGACCTCGGATCAGGTAGGAATACCCGCTGAACTTAA 516
Group08      TTGACCTCGGATCAGGTAGGAATACCCGCTGAACTTAA 516
Group02      TTGACCTCGGATCAGGTAGGAATACCCGCTGAACTTAA 515
Group03      TTGACCTCGGATCAGGTAGGAATACCCGCTGAACTTAA 514
Group05      TTGACCTCGGATCAGGTAGGAATACCCGCTGAACTTAA 515
Group11      TTGACCTCGGATCAGGTAGGAATACCCGCTGAACTTAA 515
             **************************************
```

BT

```
K     CCAGAAGGCAGCACCAATCTGGTTACCCTGTTGGATTGAAAAGACGAGAGTCAGTGTTCT 60
E     CCAGAAGGCAGCACCAATCTGGTTACCCTGTTGGATTGAAAAGACGAGAGTCAGTGTTCT 60
A     CCAGAAGGCAGCACCAATCTGGTTACCCTGTTGGATTGAAAAGACGAGAGTCAGTGTTCT 60
I     CCAGAAGGCAGCACCAATCTGGTTACCCTGTTGGATTGAAAAGACGAGAGTCAGTGTTCT 60
D     CCAGAAGGCAGCACCAATCTGGTTACCCTGTTGGATTGAAAAGACGAGAGTCAGTGTTCT 60
G     CCAGAAGGCAGCACCGATCTGGTTACCCTGTTGGATTGAAAAGACGAGAGTCAGTGTTCT 60
P     CCAGAAGGCAGCACCGATCTGGTTACCCTGTTGGATTGAAAAGACGAGAGTCAGTGTTCT 60
Q     CCAGAAGGCAGCACCGATCTGGTTACCCTGTTGGATTGAAAAGACGAGAGTCAGTGTTCT 60
J     CCAGAAGGCAGCACCGATCTGGTTACCCTGTTGGATTGAAAAGACGAGAGTCAGTGTTCT 60
R     CCAGAAGGCAGCACCGATCTGGTTACCCTGTTGGATTGAAAAGACGAGAGTCAGTGTTCT 60
C     CCAGAAGGCAGCACCGATCTGGTTACCCTGTTGGATTGAAAAGACGAGAGTCAGTGTTCT 60
B     CCAGAAGGCAGCACCGATCTGGTTACCCTGTTGGATTGAAAAGACGAGAGTCAGTGTTCT 60
O     CCAGAAGGCAGCACCGATCTGGTTACCCTGTTGGATTGAAAAGACGAGAGTCAGTGTTCT 60
M     CCAGAAGGCAGCACCGATCTGGTTACCCTGTTGGATTGAAAAGACGAGAGTCAGTGTTCT 60
H     CCAGAAGGCAGCACCGATCTGGTTACCCTGTTGGATTGAAAAGACGAGAGTCAGTGTTCT 60
F     CCAGAAGGCAGCACCGATCTGGTTACCCTGTTGGATTGAAAAGACGAGAGTCAGTGTTCT 60
Loci  ***************8********************************************

K     GACCGCACGAAAATAGAGCGCGTAATAGGCTGGACAGGAAGAGGTAGAGAGGGGAGGCAA 120
E     GACCGCACGAAAATAGAGCGCGTAATAGGCTGGACAGGAAGAGGTAGAGAGGGGAGGCAA 120
A     GACCGCACGAAAATAGAGCGCGTAATAGGCTGGACAGGAAGAGGTAGAGAGGGGAGGCAA 120
I     GACCGCACGAAAATAGAGCGCGTAATAGGCTGGACAGGAAGAGGTAGAGAGGGGAGGCAA 120
D     GACCGCACGAAAATAGAGCGCGTAATAGGCTGGACAGGAAGAGGTAGAGAGGGGAGGCAA 120
G     GACCGCACGAAAATAGAGCGCGTAATAGGCTGGACAGGAAGAGGTAGAGAGGGGAGGCAA 120
P     GACCGCACGAAAATAGAGCGCGTAATAGGCTGGACAGGAAGAGGTAGAGAGGGGAGGCAA 120
Q     GACCGCACGAAAATAGAGCGCGTAATAGGCTGGACAGGAAGAGGTAGAGAGGGGAGGCAA 120
J     GACCGCACGAAAATAGAGCGCGTAATAGGCTGGACAGGAAGAGGTAGAGAGGGGAGGCAA 120
R     GACCGCACGAAAATAGAGCGCGTAATAGGCTGGACAGGAAGAGGAAGAGAGGGGAGGCAA 120
C     GACCGCACGAAAATAGAGCGCGTAATAGGCTGGACAGGAAGAGGTAGAGAGGGGAGGCAA 120
B     GACCGCACGAAAATAGAGCGCGTAATAGGCTGGACAGGAAGAGGTAGAGAGGGGAGGCAA 120
O     GACCGCACGAAAATAGAGCGCGTAATAGGCTGGACAGGAAGAGGTAGGGAGGGGAGGCAA 120
M     GACCGCACGAAAATAGAGCGCGTAATAGGCTGGACAGGAAGAGGAAGAGAGGGGAGGCAA 120
H     GACCGCACGAAAATAGAGCGCGTAATAGGCTGGACAGGAAGAGGTAGAGAGGGGAGGCAA 120
F     GACCGCACGAAAATAGAGCGCGTAATAGGCTGGACAGGAAGAGGTAGAGAGGGGAGGCAA 120
Loci  ********************************************9**1************
                                                     0

K     AGAGACGCCATCTCCCTCACCTTCCTGCCGTCCTATTCTTCTTCCAGGGGTGGTATCAAC 180
E     AGAGACGCCATCTCCCTCACCTTCCTGCCGTCCTATTCTTCTTCCAGGGGTGGTATCAAC 180
A     AGAGACGCCATCTCCCTCACCTTCCTGCCGTCCTATTCTTCTTCCAGGGGTGGTATCAAC 180
I     AGAGACGCCATCTCCCTCACCTTCCTGCCGTCCTATTCTTCTTCCAGGGGTGGTATCAAC 180
D     AGAGACGCCATCTCCCTCACCTTCCTGCCGTCCTATTCTTCTTCCAGGGGTGGTATCAAC 180
G     AGAGACGCCATCTCCCTCACCTTCCTGCCGTCCTATTCTTCTTCCAGGGGTGGTATCAAC 180
P     AGAGACGCCATCTCCCTCACCTTCCTGCCGTCCTGTTCTTCTTCCAGGGGTGGTATCAAC 180
Q     AGAGACGCCATCTCCCTCACCTTCCTGCCGTCCTGTTCTTCTTCCAGGGGTGGTATCAAC 180
J     AGAGACGCCATCTCCCTCACCTTCCTGCCGTCCTGTTCTTCTTCCAGGGGTGGTATCAAC 180
R     AGAGACGCCATCTCCCTCACCTTCCTGCCGTCCTATTCTTCTTCCAGGGGTGGTATCAAC 180
C     AGAGACGCCATCTCCCTCACCTTCCTGCCGTCCTATTCTTCTTCCAGGGGTGGTATCAAC 180
B     AGAGACGCCATCTCCCTCACCTTCCTGCCGTCCTATTCTTCTTCCAGGGGTGGTATCAAC 180
O     AGAGACGCCATCTCCCTCACCTTCCTGCCGTCCTATTCTTCTTCCAGGGGTGGTATCAAC 180
M     AGAGACGCCATCTCCCTCACCTTCCTGCCGTCCTATTCTTCTTCCAGGGGTGGTATCAAC 180
H     AGAGACGCCATCTCCCTCACCTTCCTGCCGTCCTATTCTTCTTCCAGGGGTGGTATCAAC 180
F     AGAGATGCCATCTCCCTCACCTTCCTGCCGTCCTATTCTTCTTCCAGGGGTGGTATCAAC 180
Loci  *****1****************************1*************************
           1                            2
```

```
K           TTACGCACTGGCCGGTCTGGAGGTGAACCTATTTGAGAGGGGACAAACACGTCGTTAGCT 240
E           TTACGCACTGGCCGGTCTGGAGGTGAACCTATTTGAGAGGGGACAAACACGTCGTTAGCT 240
A           TTACGCACTGGCCGGTCTGGAGGTGAACCTATTTGAGAGGGGACAAACACGTCGTTAGCT 240
I           TTACGCACTGGCCGGTCTGGAGGTGAACCTATTTGAGAGGGGACAAACACGTCGTTAGCT 240
D           TTACGCACTGGCCGGTCTGGAGGTGAACCTATTTGAGAGGGGACAAACACGTCGTTAGCT 240
G           TTACGCACTGGCCGGTCTGGAGGTGAACCTATTTGAGAGGGGACAAACACGTCGTTAGCT 240
P           TTACGCACTGGCCGGTCTGGAGGTGAACCTATTTGAGAGGGGACAAACACGTCGTTAGCT 240
Q           TTACGCACTGGCCGGTCTGGAGGTGAACCTATTTGAGAGGGGACAAACACGTCGTTAGCT 240
J           TTACGCACTGGCCGGTCTGGAGGTGAACCTATTTGAGAGGGGACAAACACGTCGTTAGCT 240
R           TTACGCACTGGCCGGTCTGGAGGTGAACCTATTTGAGAGGGGACAAACACGTCGTTAGCT 240
C           TTACGCACTGGCCGGTCTGGAGGTGAACCTATTTGAGAGGGGACAAACACGTCGTTAGCT 240
B           TTACGCACTGGCCGGTCTGGAGGTGAACCTATTTGAGAGGGGACAAACACGTCGTTAGCT 240
O           TTACGCACTGGCCGGTCTGGAGGTGAACCTATTTGAGAGGGGACAAACACGTCGTTAGCT 240
M           TTACGCACTGGCCGGTCTGGAGGTGAACCTATTTGAGAGGGGACAAACACGTCGTTAGCT 240
H           TTACGCACTGGCCGGTCTGGAGGTGAACCTATTTGAGAGGGGACAAACACGTCGTTAGCT 240
F           TTACGCACTGGCCGGTCTGGAGGTGAACCTATTTGAGGGGGGAAAACACGTCGTTAGCA 240
Loci        *************************************1*****1***************1
                                                 3     4               5

K           AAAGATTCGACTGACGCAAAGCTGCGAGACCCTTTCTCGTTTCTCCTCGCCTGTT--CCC 300
E           AAAGATTCGACTGACGCAAAGCTGCGAGACCCTTTCTCGTTTCTCCTCGCCTGTT---CC 300
A           AAAGATTCGACTGACGCAAAGCTGCGAGACCCTTTCTCGTTTCTCCTCGCCTGTT-CCCC 300
I           AAAGATTCGACTGACGCAAAGCTGCGAGACCCTTTCTCGTTTCTCCTCGCCTGTTCCCCC 300
D           AAAGATTCGACTGACGCAAAGCTGCGAGACCCTTTCTCGTTTCTCCTCGCCTGTT-CCCC 300
G           AAAGATTCGACTGACGCAAAGCTGCGAGACCCTTTCTCGTTTCTCCTCGCCTGTT-CCCC 300
P           AAAGATTCGACTGACGCAAAGCTGCGAGACCCTTTCTCGTTTCTCCTCGCCTGTTT-CCC 300
Q           AAAGATTCGACTGACGCAAAGCTGCGAGACCCTTTCTCGTTTCTCCTCGCCTGTT--CCC 299
J           AAAGATTCGACTGACGCAAAGCTGCGAGACCCTTTCTCGTTTCTCCTCGCCTGTT-CCCC 300
R           AAAGATTCGACTGACGCAAAGCTGCGAGACCCTTTCTCGTTTCTCCTCGCCTGTT--CCC 299
C           AAAGATTCGACTGACGCAAAGCTGCGAGACCCTTTCTCGTTTCTCCTCGCCTGTT---CC 298
B           AAAGATTCGACTGACGCAAAGCTGCGAGACCCTTTCTCGTTTCTCCTCGCCTGTT--CCC 299
O           AAAGATTCGACTGACGCAAAGCTGCGAGACCCTTTCTCGTTTCTCCTCGCCTGTT--CCC 299
M           AAAGATTCGACTGACGCAAAGCTGCGAGACCCTTTCTCGTTTCTCCTCGCCTGTT-CCCC 300
H           AAAGATTCGACTGACGCAAAGCTGCGAGACCCTTTCTCGTTTCTCCTCGCCTGTT-CCCC 300
F           AAAGATTCGACTGACGCAAAGCTGCGAGACCCTTTCTCGTTTCTCCTCGCCTGT--CCCC 300
Loci        ******************************************************1111**
                                                                  6789

K           CCCCCTTTTTGGGTTGGAATGTTGACTTGGGTGCACGTTGGCCGGCGGGGTAATAGAAAAGAG 361
E           CCCCCTTTTTGGGTTGGAATGTTGACTTGGGTGCACGTTGGCCGGCGGGGTAATAGAAAAGAG 360
A           CCCCCTTTTTGGGTTGGAATGTTGACTTGGGTGCACGTTGGCCGGCGGGGTAATAGAAAAGAG 362
I           CCCCCTTTTTGGGTTGGAATGTTGACTTGGGTGCACGTTGGCCGGCGGGGTAATAGAAAAGAG 363
D           CCCCCTTTTTGGGTTGGAATGTTGACTTAGGTGCACGTTGGCCGGCGGGGTAATAGAAAAGAG 362
G           CCCCCTTTTTGGGTTGGAATGTTGACTTAGGTGCACGTTGGCCGGCGGGGTAATAGAAAAGAG 362
P           CCCCCTTTTTGGGTTGGAATGTTGACTTGGGTGCACGTTGGCCGGCGGGGTAATAGAAAAGAG 362
Q           CCCCCTTTTTGGGTTGGAATGTTGACTTGGGTGCACGTTGGCCGGCGGGGTAATAGAAAAGAG 361
J           CCCCCTTTTTGGGTTGGAATGTTGACTTGGGTGCACGTTGGCCGGCGGGGTAATAGAAAAGAG 362
R           CCCCCTTTTTGGGTTGGAATGTTGACTTGGGTGCACGTTGGCCGGCGGGGTAATAGAAAAGAG 361
C           CCCCCTTTTTGGGTTGGAATGTTGACTTGGGTGCACGTTGGCCGGCGGGGTAATAGAAAAGAG 360
B           CCCCCTTTTTGGGTTGGAATGTTGACTTGGGTGCACGTTGGCCGGCGGGGTAATAGAAAAGAG 361
O           CCCCCTTTTTGGGTTGGAATGTTGACTTGGGTGCACGTTGGCCGGCGGGGTAATAGAAAAGAG 361
M           CCCCCTTTTTGGGTTGGAATGTTGACTTGGGTGCACGTTGGCCGGCGGGGTAATAGAAAAGAG 362
H           CCCCCTTTTTGGGTTGGAATGTTGACTTGGGTGCACGTTGGCCGGCGGGGTAATAGAAAAGAG 362
F           CCCCCTTTTTGGGTTGGAATGTTGACTTGGGTGCACGTTGGCCGGCGGGGTAATAGAAAAGAG 361
Loci        ****************************2**********************************
                                        0
```

MAP

```
α      GGGACTGCTATCAATTTGCTGATTGGATATATACCAGGCCTACCCTTCTCCTCTCAACTA 60
δ      GGGACTACTATCAATTTGCTGATTGGATATATACCAGGCCTACCCTTCTCCTCTCAACTA 60
β      GGGACTGCTATCAATTTGCTGATTGGATATATACCAGGCCTACCCTTCTCCTCTCAACTA 60
γ      GGGGCTGCTATCAATTTGCTGATTGGATATATACCAGGCCTACCCTTCTCCTCTCAACTA 60
Loci   ***2**2*****************************************************
          1  2

α      CAACAATTTCCCCAAGTCTTGTTGTACATCGATAAACGAAGTCATTTGCCACGGCATTCC 120
δ      CAACAATTTCCCCAAGTCTTGTTGTACATCGATAAACGAAGTCATTTGCCACGGCATTCC 120
β      CAACAATTTCCCCAAGTCTTGTTGTACATCGATAAACGAAGTCATTTGCCATGGCATTCC 120
γ      CAACAATTTCCCCAAGTCTTGTTGTACATCGATAAACGAAGTCATTTGCCACGGCATTCC 120
Loci   ***************************************************2********
                                                          3

α      CGACCAGCGAGTCCTCCTCGACGGAGACATCCTCAATATCGACGTTTCCCTCTACCACGA 180
δ      CGACCAGCGAGTCCTCCTCGACGGAGACATCCTCAATATCGACGTTTCCCTCTACCATGA 180
β      CGACCAGCGAGTCCTCCTCGACGGAGACATCCTCAATATCGACGTTTCCCTCTACCACGA 180
γ      CGACCAGCGAGTCCTCCTCGACGGAGACATTCTCAATATCGACATTTCCCTCTACCACGA 180
Loci   ******************************2************2*************2**
                                     4            5             6

α      AGGCTACCATGCCGATTTGAATGAGACATACTACATTGGCGACAAGGCCAAAGCAGATCC 240
δ      AGGCTACCATGCCGATTTGAATGAGACATACTACATTGGCGACAAGGCCAAAGCAGATCC 240
β      AGGCTACCATGCCGATTTGAATGAGACATACTACATTGGCGACAAGGCCAAAGCAGATCC 240
γ      AGGCTACCACGCCGATTTGAATGAGACATACTACATTGGCGACAAGGCCAAAGCAGATCC 240
Loci   *********2**************************************************
                7

α      CGATAGTGTGCGCGTCGTCGAGGCGGCCCGTGAGTGCCTGGAAGAATCCATCAAGGCCGT 300
δ      CGATAGTGTGCGCGTCGTCGAGGCGGCCCGTGAGTGCCTGGAAGAATCCATCAAGGCCGT 300
β      CGATAGTGTGCGCGTCGTCGAGGCGGCCCGTGAGTGCCTGGAAGAATCCATCAAGGCCGT 300
γ      CGATAGTGTGCGCGTCGTCGAGGCGGCCCGTGAGTGCCTGGAAGAATCCATCAAGGCCGT 300
       ************************************************************

α      CAAGCCGGGCACCTTGATCCGTGAATTCGGAAACATCATTGAGAAGCATGCCAAGGCAAA 360
δ      CAAGCCGGGCACCTTGATCCGTGAATTCGGAAACATCATTGAGAAGCATGCCAAGGCAAA 360
β      CAAGCCGGGCACCTTGATCCGTGAATTCGGAAACATCATTGAGAAGCATGCCAAGGCAAA 360
γ      CAAGCCGGGCACCTTGATCCGTGAATTCGGAAACATCATTGAGAAGCATGCCAAGGCAAA 360
       ************************************************************

α      GAATT 365
δ      GAATT 365
β      GAATT 365
γ      GAATT 365
       *****
```
