# Supplementary figures and images for: Population Structure of Geosmithia morbida, the Causal Agent of Thousand Cankers Disease of Walnut Trees in the United States
Source: PLoS One. 2014 Nov 13;9(11):e112847. doi: 10.1371/journal.pone.0112847 (PMC4231075; doi:10.1371/journal.pone.0112847)

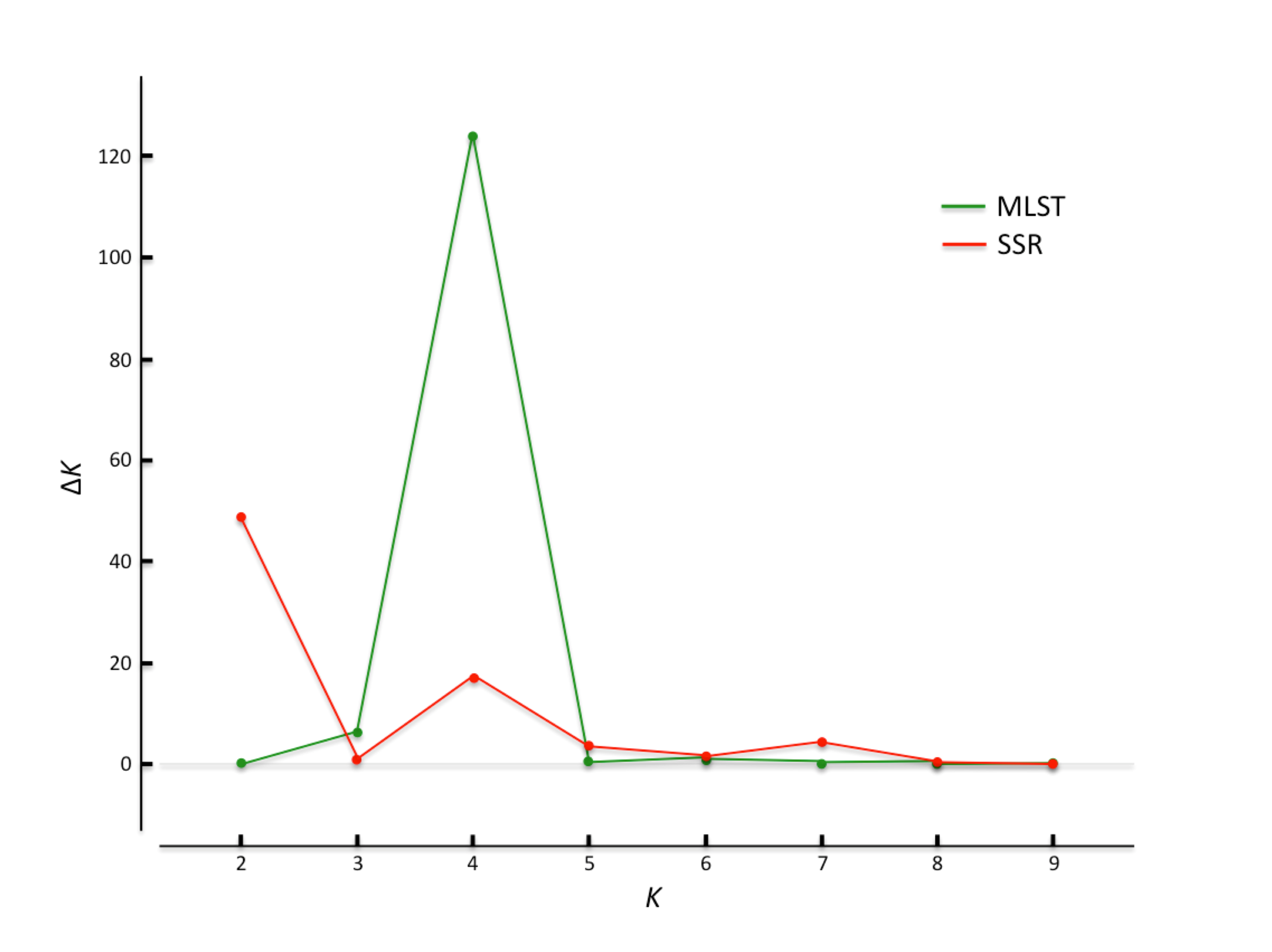

Supplement: Figure S2 — Results of Δ K computation for Geosmithia morbida isolates. Scenario where K = 1-10 (20 iterations) by using STRUCTURE [39]. SSR data supports clusters of 2 and 4, whereas MLST data supports a cluster of 4. (TIF) [file pone.0112847.s002.tif]
