# Supplementary material for: Population Structure of Geosmithia morbida, the Causal Agent of Thousand Cankers Disease of Walnut Trees in the United States
Source: PLoS One. 2014 Nov 13;9(11):e112847. doi: 10.1371/journal.pone.0112847 (PMC4231075; doi:10.1371/journal.pone.0112847)
Supplement: Table S1 — Microsatellite (SSR) loci used to characterize Geosmithia morbida isolates. (PDF) [file pone.0112847.s003.pdf]

SSR name	GenBank identification	Motif(s)	Number of Alleles	Size (bp)	Primers
Geo4045	KF574259	CT	4	182 - 195	TCGGTGAATTGATCGGTGTA
					CTCCCAGCCGTACTAACGAC
Geo223	KF574250	CT	4	175 - 185	ACGTCACAGGAGGGAGAGAA
					CGTGGTGGTTCTCGTAACAT
Geo3896	KF574256	CT	5	200 - 226	CGGGTAGGGTGACCACGA
					GTGGTCGAGGACAACAAGGT
Geo3416	KF574255	GT	5	214 - 244	AGTTCGCGAAAGAGCACAAT
					GTTCACACAGAAGGTTGTTAGTATGG
Geo7713	KF574258	CA	11	293 - 411	CGTACGATGGTATTTATCTCATTCA
					TTACACGTAGGATGGCATGG
Geo194	KF574251	GT/AG	7	253 - 285	CCCACGTCAAGTCTGTCA
					TCCTCCTCAGTCGCATACTCT
Geo2849	KF574254	GA/AAT	4	310 - 341	CGCCTACGGAATACCTACC
					AGGACGAAACATCACCGAGT
Geo2514	KF574253	AC/TA	4	259 - 280	TTAACATGGCCAACACAACC
					TCATTGATGTCGGCAAAGAC
Geo1851	KF574252	AGC	6	202 - 228	GGGCAATATAACGGTTT
					CCTCGTAGGCGTAAGTCTGG
Geo6823	KF574257	TTG/GA	9	303 - 331	TTCAGCATCATTACTTGTTGG
					TCGTCGTCGTTGCTGTAGTC
