# Supplementary material for: Population Structure of Geosmithia morbida, the Causal Agent of Thousand Cankers Disease of Walnut Trees in the United States
Source: PLoS One. 2014 Nov 13;9(11):e112847. doi: 10.1371/journal.pone.0112847 (PMC4231075; doi:10.1371/journal.pone.0112847)
Supplement: File S1 — Development of MLST-primers based on partial sequencing of the Geosmithia morbida (CBS 124663) genome. (DOCX) [file pone.0112847.s006.docx]

**Development of MLST-primers based on partial sequencing of the *Geosmithia morbida* (CBS 124663) genome**

High-quality total DNA of *G. morbida* isolate 1217 (CBS 124663) was extracted as described in “DNA extraction” (Materials and Methods) and skim-sequenced at the Purdue University Genomics Core Facility. A half-plate run on a 454 FLX standard pyrosequencing [[1](#_ENREF_1)] of a 3 kb-long paired end library generated 779,553 reads representing 116,376,601 bp. Sequences were assembled in Newbler 2.3 (454 Life Sciences Corp, CT, USA) by using default settings. This resulted in the assembly of 628,023 reads (80.56% of the total), which were organized into 27,933 contigs, representing 15,924,440 bp of the *G. morbida* genome. A total 24,982 sequences (contigs and singletons) larger than 200 bp were automatically annotated with the Blast2GO similarity tool [[2](#_ENREF_2)], and 92% were homologous to predicted proteins deposited in the NR database (BlastX, e-value cut-off = 10^-5^); 81% had a GO term [[3](#_ENREF_3)] associated with their hit, and 61% were homologous to a predicted protein with known function. Six contigs containing the partial sequences of predicted housekeeping genes (Table 2) were selected and primers flanking these sequences were designed. The sequences were: methionine aminopeptidase (KF947520); ribosomal L18ae protein family (KF947521); dolichyl-phosphate-mannose-protein mannosyltransferase (KF947522); amino acid permease (KF947523); 40S ribosomal protein S2 (KF947524); and kinesin (KF947525). Primers were used for the establishment of a MLST scheme for *G. morbida*.

**References**

1. Margulies M, Egholm M, Altman WE, Attiya S, Bader JS, et al. (2005) Genome sequencing in microfabricated high-density picolitre reactors. Nature 437: 376-380.

2. Conesa A, Götz S, García-Gómez JM, Terol J, Talón M, et al. (2005) Blast2GO: a universal tool for annotation, visualization and analysis in functional genomics research. Bioinformatics 21: 3674-3676.

3. Ashburner M, Ball CA, Blake JA, Botstein D, Butler H, et al. (2000) Gene Ontology: tool for the unification of biology. Nature Genetics 25: 25-29.
